# Improving the resolution of solar energy potential maps derived from global DSMs for rooftop solar panel placement using deep learning

**DOI:** 10.1016/j.heliyon.2024.e41193

**Published:** 2024-12-13

**Authors:** Maryam Hosseini, Hossein Bagheri

**Affiliations:** Faculty of Civil Engineering and Transportation, University of Isfahan, Isfahan, Iran

**Keywords:** Solar energy map, Super-resolution, Digital elevation model, Deep learning, Solar panel placement

## Abstract

This study focuses on generating high-resolution annual solar energy potential maps (ASMs) using global Digital Elevation Models (DEMs) to aid in solar panel placement, especially in urban areas. A framework was developed to enhance the resolution of these maps. Initially, the accuracy of ASMs derived from various DEMs was compared with LiDAR-derived ASMs. The evaluations indicated that the Copernicus DEM provided a highly accurate ASM. Subsequently, deep learning algorithms were trained to improve the resolution of the LiDAR-derived ASM. The results demonstrated that the Enhanced Deep Super-Resolution (EDSR) Network outperformed the U-Net-based model. The trained EDSR model was then utilized to enhance the resolution of the Copernicus ASM. Comparing the enhanced-resolution map of Copernicus respective to LiDAR showed that the EDSR model provided the necessary generalizability to improve the accuracy and resolution of the Copernicus ASM, particularly in urban areas. The investigations revealed that the improved resolution map with a resolution of 6 m, achieving RMSE of 35.75 $\frac{MWh}{m^{2}}$ and a correlation of 0.87 respective to LiDAR data, was capable of locating solar panels on buildings, whereas the original Copernicus-derived maps with a 30 m resolution had RMSE of 51.26 $\frac{MWh}{m^{2}}$ and a correlation of 0.72 for such placement purposes.

## 1 Introduction

Fossil fuels such as coal, natural gas, and oil are the primary energy sources in the global economy. However, using fossil fuels has significant negative consequences, including the emission of greenhouse gases and other pollutants, leading to increased air pollution [3]. A key solution to mitigate the adverse impacts of fossil fuels is to improve energy efficiency and switch to renewable sources [34], like solar energy, which is unlimited and clean [41].

Solar photovoltaic (PV) systems are the primary form of harnessing solar energy [37], [38]. However, there are challenges that hinder the widespread adoption of PV systems. One significant limitation is that PV systems require a large area for installation. As land costs increase over time, particularly in urban areas, the demand for efficient land utilization increases [41].

To address space constraints and high land costs, integrated PV systems can be installed on buildings in densely populated urban areas [18], utilizing rooftops and facades. Estimating the solar energy potential of these buildings is crucial for future energy planning [14]. Sound barriers near roads, railways, and highway margins are also suitable for PV installations due to their accessibility, minimal shading effects of vegetation [24], [81], and lower risk of complaints or environmental damage [23], [41]. Choosing appropriate locations for solar panels in both urban and non-urban areas is essential for maximizing solar energy as a renewable source.

The amount of solar radiation dramatically influences the efficiency of PV systems. Understanding the spatial distribution of solar radiation is vital in designing and installing these systems. Factors like elevation, surface orientation, and shadows created by topographic features significantly affect the received solar radiation, especially in areas with complex topography [82].

Assessing the solar energy potential through solar energy potential maps is a valuable analytical tool for assessing local energy generation capabilities. These maps help design and implement energy strategies aligned with sustainable development goals. Understanding the capacity of installed panels and identifying the best locations for solar panel installation can significantly enhance the expansion of renewable energy production [70].

The generation of solar energy potential maps typically relies on ground measurements from meteorological and climatic stations. However, these measurements are point-based, necessitating spatial interpolation to create cohesive maps. Various interpolation techniques, like spline functions [36], [69], [85], [6], weighted averaging, and kriging [6], [35], [88], are used to estimate solar potential, especially in flat areas with uniform climates [72]. However, their reliability declines in regions with complex topography due to factors like snow cover and cloud effects on radiation [72]. Moreover, these methods require a high density of measurement stations, which is often not feasible, particularly in urban areas. The quality of interpolation decreases significantly in complex terrains and may not account for all factors influencing solar energy estimation, such as shading from nearby topographic features [55].

Continuous radiation values can be obtained from stationary weather satellites, offering extensive spatial coverage, though they are less accurate than ground measurements, particularly in cloudy conditions. For instance, the second-generation Meteosat provides data every 15 minutes with a 2.5 km spatial resolution at Nadir. Despite improvements, satellite data still struggle with accuracy in complex terrains and urban areas due to low spatial resolution [82]. While building and maintaining ground stations is expensive, and satellite measurements have relatively high spatio-temporal resolution, solar radiation models offer a cost-effective alternative, especially in areas with complex topography. These models use digital elevation data, including slope and aspect, to efficiently estimate solar radiation across large regions.

A Digital Elevation Model (DEM) is a virtual representation of the Earth's surface that describes its elevation. DEMs are essential in various fields like hydrological modeling, meteorology, climate modeling, and solar radiation modeling. They provide critical data on absolute surface elevations and derived parameters like slope and aspect, which affect both the incoming solar radiation and outgoing longwave radiation, particularly in areas with complex topography [83]. DEMs help estimate the horizon and the sun's relative position at any given time, which is crucial for calculating direct and diffuse solar radiation using various models [82]. Several software packages, such as Arc GIS (SolarFlux [21], [30], Solar Analyst [25]), SRAD [84], IDIRISI (Solei) [56], and GRASS (r.sun) [32], are available to model solar radiation using DEM data. Numerous studies have evaluated the accuracy of these models in complex terrains [82], [66], [52], [11]. These models vary in their approaches but generally use topographic information from the DEM, such as elevation, surface orientation, and shadow effects, to estimate solar radiation at each point on the surface.

A major technical limitation in previous studies on solar energy potential is the low spatial resolution of solar maps [47], [77]. High resolution solar maps reduce uncertainty in detailed analyses, particularly in small or complex regions [41]. DEMs with different resolutions can produce varying estimates of elevation, slope, aspect, and shadow effects, leading to differences in solar radiation estimations, especially in complex terrains [7], [5], [8], [16]. Research has shown that the resolution and vertical accuracy of DEMs significantly impact the accuracy of solar energy potential estimates. For example, a study in Sichuan Province, China, using various DEMs with different resolutions, found that while the absolute height values were similar, their derivatives (slope and aspect) differed, affecting solar energy estimations [83]. Another study in the North Pole's ice cap region revealed that using a coarse-resolution DEM (2000 m) led to overestimating of both direct and total solar energy potential [2]. It should be noted that meteorological conditions affect the estimation of solar radiation in addition to the spatial resolution of DEMs [57].

DEMs can be generated through various methods, including ground-based surveying [6] and satellite-based remote sensing techniques such as satellite stereo images [4], [10] and airborne LiDAR [14]. Ground-based DEM acquisition, while accurate, is costly and time-consuming, especially for large or inaccessible areas with complex topography. DEM derivatives like slope and aspect are crucial in determining solar energy potential. High resolution DEMs result in more precise solar energy potential maps [83]. Remote sensing methods like LiDAR enable the creation of highly accurate, high resolution DEMs, making it feasible to generate precise solar energy potential maps, particularly in urban areas.

Despite developing sensors such as LiDAR and the ability to generate high resolution DEMs, these models are unavailable in all regions worldwide. Just for some parts of Europe [42], [58] and America, Free LiDAR data is available. Furthermore, LiDAR data requires expensive equipment, comes in a point cloud format, and has a high processing volume. It also has less spatial coverage compared to satellite data. To reduce the cost of producing high resolution maps, various machine learning techniques have been implemented to improve the spatial resolution of solar energy potential maps [47], [77]. Since machine learning is a statistical approach to prediction, it is not limited to human understanding of the problem and does not require it.

Deep learning algorithms, recognized as state-of-the-art in machine learning, have been increasingly used to enhance the resolution of meteorological parameters [28], [62], [78], such as temperature and precipitation. However, there has been limited research on improving the spatial resolution of solar radiation. These studies have focused on enhancing solar radiation resolution at very low resolutions for meteorological and climate applications. For instance, Stengel et al. (2020) proposed a deep learning adversarial approach to increase the resolution of wind speed data by 50 times, from a spatial resolution of 100 km to 2 km, as well as increase the resolution of solar radiation outputs from global weather models by 25 times, from a spatial resolution of 100 km to 4 km [77]. Another study by Rupa Kurinchi-Vendhan et al. (2021) investigated several deep learning techniques, such as Enhanced Super-Resolution Generative Adversarial Network (ESRGAN), Enhanced Deep Super-Resolution Network (EDSR), Physics-Informed Resolution-Enhanced Generative Adversarial Network (PhIREGAN), and Super-Resolution Convolutional Neural Network (SRCNN), for five times resolution improvement of wind speed and solar radiation outputs from global weather models with a spatial resolution of 10 km to 2 km. They demonstrated the effectiveness of these methods in improving resolution [47].

Therefore, previous studies have shown that deep learning models can enhance the resolution of solar radiation at low resolutions, suggesting that, in general, increasing the resolution in this field is feasible [77], [47]. However, most research has focused on tiny-scale resolution improvement for climate and meteorological applications. At this scale, due to the very low resolution of the products, the overall trend of solar energy variations is captured. However, they lack the detailed information needed for energy resource management, especially in urban areas with tall buildings that obstruct sunlight. This changes the potential of solar energy on rooftops of buildings in urban areas or regions with complex topography [1]. Higher resolution maps are necessary to accurately assess solar energy potential on building rooftops in such areas. These detailed maps are crucial for applications such as the placement of solar panels and the location of solar power plants, where precise energy potential data is essential for effective energy management and investment decisions.

One of the useful resources for generating solar energy potential maps is DEMs with medium resolution. These DEMs are generally available for most locations on Earth either for free (such as ASTER, ALOS, SRTM and Copernicus) or at a low cost (such as TanDEM-X and Cartosat). Due to their extensive spatial coverage, they enable the production of solar energy potential maps in most less developed areas with lower costs. However, the low resolution of these DEMs compared to those obtained from LiDAR data reduces the accuracy of extracting solar energy potential maps, which poses limitations for precise location placement of solar panels, especially in urban areas and surroundings of highways. A proposed solution to increase the resolution of solar energy maps derived from medium resolution DEMs for more accurate solar panel placement, particularly in urban areas, is the utilization of deep learning techniques. Therefore, it is necessary to investigate the capability of these algorithms to enhance the resolution for the mentioned applications. One of the objectives of this study is to investigation the potential of deep learning networks for improving the resolution of solar energy maps obtained from medium resolution DEMs to achieve higher details, especially in urban areas, for applications such as locating PV panels. In the proposed method in this paper first, the capability of deep learning networks in enhancing the resolution of solar energy maps derived from LiDAR is explored. After training the network and acquiring the necessary ability to improve the resolution, it is used to increase the resolution of solar energy potential maps obtained from medium resolution DEMs. Hence, another goal of this research is to investigate the transferability of deep learning networks for resolution enhancement of solar energy maps obtained from medium resolution DEMs. Considering the lack of access to LiDAR data and its high cost for the general public, especially in developing or less developed regions, and the increasing demand for solar energy utilization in these areas, the use of pre-trained models and transfer learning to freely accessible DEMs enable more accurate, and high resolution estimation of solar energy worldwide. This study also evaluates the accuracy of freely available DEMs such as SRTM, ASTER, Copernicus, and ALOS, in estimating solar energy potential compared to LiDAR. This evaluation is conducted for various land uses, including urban areas (on rooftop surfaces for solar panel installation and the potential amount of generated electrical energy), non-urban areas for solar power plant siting, and areas alongside highways. Furthermore, the capability of improved solar energy maps in locating solar panels on building surfaces is evaluated.

In summary, the main contributions of this research lie in two categories: 1) Training the deep learning-based super-resolution models on LiDAR derived ASMs and transferring its learning to enhance the spatial resolution of global DEM derived ASM, for which we had no high resolution patches available, 2) Evaluating the results of super-resolution for accurate solar panel placement localization, in different land types particularly in urban areas.

This paper consists of several sections. The research objectives and a review of previous studies have been introduced earlier. The study area and the data used in this study are presented in Sections 2 and 3, respectively. In Section 4, the proposed framework for improving the resolution of solar energy maps is described. Then, the results and conducted evaluations are presented in Section 5. In Section 6, the obtained results and the capabilities of the resolution enhancement models are discussed.

## 2 Study area

**Figure 1:**
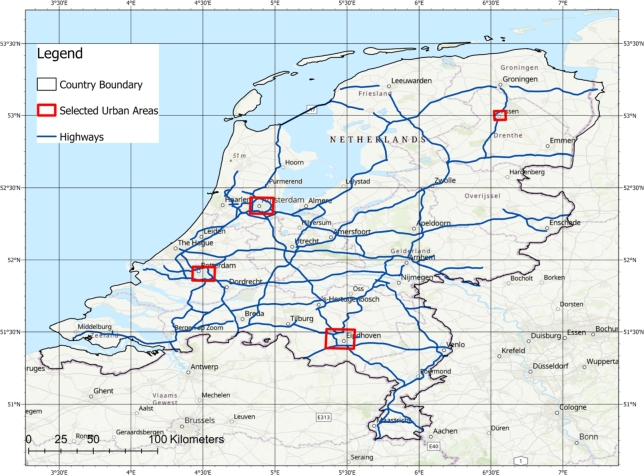
Map of the study area, the territory of the Netherlands, along with the boundaries of its important cities indicated by red rectangles. Additionally, the highways are depicted as blue lines.

Fig. 1 represents the study area of this investigation, covering the territory of the Netherlands. Situated in northwestern Europe, this country is located between the geographical latitudes of $50^{\circ }$ and $54^{\circ }$ North, and the longitudes of $3^{\circ }$ and $8^{\circ }$ East. It encompasses a total area of 41,543 square kms. The Netherlands shares its eastern border with Germany, its southern border with Belgium, and its northern and western borders with the coastal line of the North Sea. Furthermore, it has maritime boundaries in the North Sea with Britain, Germany, and Belgium.

The Netherlands consists of 12 provinces, with Amsterdam being its capital city. Rotterdam, the second-largest city in the Netherlands, is renowned for having the largest port in Europe in terms of both area and the number of docks it possesses. Other significant cities in this country include The Hague (Den Haag) and Eindhoven. The Netherlands is a predominantly low-lying and flat country, with approximately 26% of its land area and 21% of its population residing below sea level. About 50% of the Dutch territory is situated just one meter above sea level. The majority part of the Netherlands is flat, except some hills in the southeastern region and a hilly area known as the “Veluwe” in the central part of the country. The highest hills, located in the southeast, have elevations of less than 321 m above sea level.

The climate of the Netherlands is influenced by the North Sea and the Atlantic Ocean, resulting in cool, cloudy, and humid conditions for most of the year. Sunlight is scarce in the Netherlands from November to February. The maximum amount of sunshine during the day is around 6-7 hours, often accompanied by rainfall [17].

In Fig. 1, in addition to the territory of the Netherlands, the important cities, including Amsterdam, Rotterdam, Eindhoven, and Assen are indicated by red polygons. Furthermore, the major highways, that have been assessed in this study for the placement of solar panels, are depicted by blue lines.

## 3 Dataset

In this research, global DEMs available to the public, free of charge, such as SRTM, ALOS, ASTER, and Copernicus, have been utilized to estimate solar energy potential. These data are used to estimate the annual average solar energy potential.

The Advanced Spaceborne Thermal Emission and Reflection Radiometer (ASTER) is a Japanese sensor installed on the Terra satellite, launched in 1999 by NASA [13], [59]. Since February 2000, ASTER has collected satellite images in 14 bands, covering the visible and thermal infrared spectrum. These images generate detailed maps of surface temperature, emissivity, reflectance, and elevation above sea level. In July 2009, the first version of the ASTER Global DEM (ASTER GDEM) was created, offering 99% coverage of the Earth's surface (from $83^{\circ }$ South to $83^{\circ }$ North latitudes). Subsequent versions were released in October 2011 and August 2019, improving accuracy. The third version of ASTER GDEM, based on imagery from 2013 [60], was used in this study.

The Shuttle Radar Topography Mission (SRTM) was a global radar mapping mission launched by the United States National Geospatial-Intelligence Agency (NGA) and NASA [1], [73]. Conducted in an 11-day mission on February 2000, it aimed to produce DEMs covering latitudes between $56^{\circ }$ South and $60^{\circ }$ North using a synthetic aperture radar system onboard the Space Shuttle Endeavour. Until 2015, the raw SRTM data was available with a spatial resolution of one arc-second (approximately 30 m) for the United States and Australia and a spatial resolution of three arc-seconds (approximately 90 m) for the rest of the world. The third version of the SRTM DEM, used in this study, is void-filled using elevation data from ASTER Global Digital Elevation Model 2 (GDEM2), USGS Global Multi-resolution Terrain Elevation Data (GMTED) 2010, and USGS National Elevation Dataset (NED).

The Advanced Land Observing Satellite (ALOS) was launched on January 24, 2006, from the Tanegashima Space Center [80]. ALOS was one of the largest Earth observation satellites in the world, and it collected high-resolution global Earth observation data. ALOS had three remote sensing instruments, including the Panchromatic Remote-sensing Instrument for Stereo Mapping (PRISM) for DEM generation, the Advanced Visible and Near Infrared Radiometer Type-2 (AVNIR-2) provided detailed land coverage observations, and the Phased Array type L-band Synthetic Aperture Radar (PALSAR) for all weather conditions, day-and-night land observation. The ALOS global DEM (AW3D30) generated from PRISM images collected between 2006 and 2011 is available for free from the Japan Aerospace Exploration Agency (JAXA) with a horizontal resolution of one arc-second (approximately 30 m) for free [74].

The Copernicus DEM is available in three versions: EEA-10, GLO-30, and GLO-90, based on radar satellite data from the TanDEM-X mission conducted between 2011 and 2015, which became available in 2019. The TanDEM-X mission was a public-private partnership between the German government, represented by the German Aerospace Center (DLR), and Airbus Defense and Space. The EEA-10 dataset, with a spatial resolution of 10 meters, covers only 6 million square km of the Earth's surface, which includes 39 European countries and all their islands (except for French overseas territories). The GLO-30 and GLO-90 datasets provide global coverage with spatial resolutions of 30 m and 90 m, respectively, covering 149 million square km of the Earth's surface [27].

In this study, in addition to the global DEMs mentioned above, the DEM derived from LiDAR data of the Netherlands was also utilized for evaluations and training deep learning-based algorithms. This DEM is obtained from the third version of the Actueel Hoogtebestand Nederland (AHN3), which is collected by the Publieke Dienstverlening Op de Kaart (PDOK) and made freely available to the public. The AHN3 DEM is a raw raster with a 0.5 m grid spacing, where both ground and non-ground objects (such as trees, buildings, bridges, and other structures) are sampled from the point cloud to a 0.5-meter grid spacing without further editing. The AHN3 data were generated between 2014 and 2019 [61].

Table 1 displays the specifications of the various DEMs used in this study. The global DEMs have a spatial resolution of 1 arc-second (approximately 30 m) and are represented in the WGS 84 horizontal coordinate system. The SRTM, ASTER, and ALOS DEMs have the EGM96 vertical reference, while the Copernicus DEM has the EGM2008 vertical reference. The LiDAR DEM with a spatial resolution of 0.5 m has the amersfort RD-New horizontal coordinate system and the Normaal Amsterdams Peil (NAP height) vertical reference.

**Table 1:** Characteristics of DEMs used in this study, covering the Netherlands.

DEM	Spatial Resolution	Horizontal Reference	Vertical Reference	Production Year
SRTM	1 arc-second (30 m)	WGS 84	EGM96 GEOID	2000
ALOS	1 arc-second (30 m)	WGS 84	EGM96 GEOID	2006-2011
ASTER	1 arc-second (30 m)	WGS 84	EGM96 GEOID	2000-2013
Copernicus	1 arc-second (30 m)	WGS 84	EGM2008 GEOID	2011-2015
LiDAR	0.5 m	Amersfoort	Normaal Amsterdams Peil (NAP height)	2014-2019

**Figure 2:**
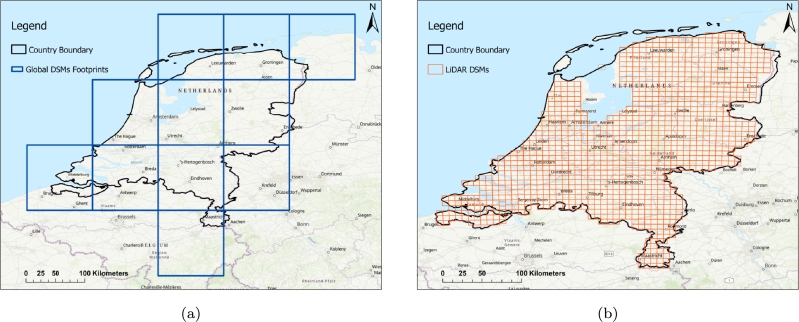
a) Visualizing the extent of global DEM tiles (SRTM, ASTER, ALOS, Copernicus) covering the study area, b) the extent of LiDAR DEM tiles over the Netherlands.

Fig. 2.a shows the extent of the global DEM tiles, and Fig. 2.b shows the extent of the LiDAR DEM tiles used in this study, covering the study area of the Netherlands.

## 4 Method

**Figure 3:**
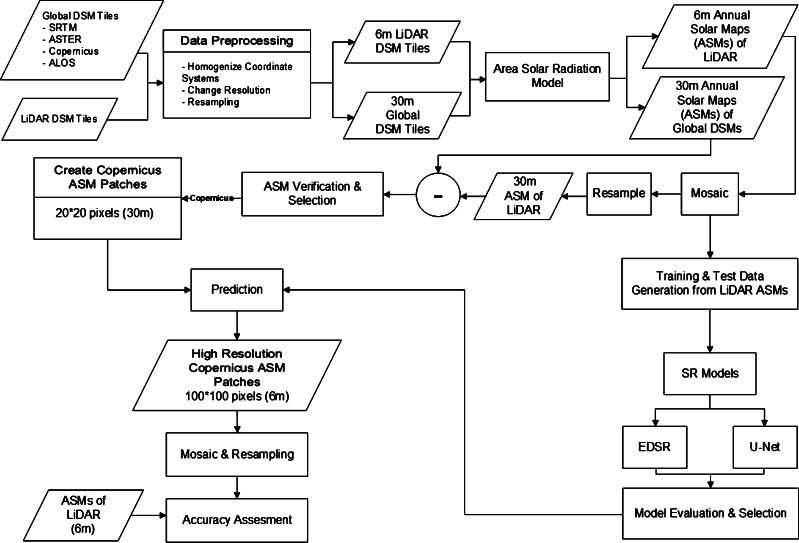
The proposed framework for super-resolution of ASMs derived from global DEMs.

Fig. 3 illustrates the proposed framework for generating and super-resolution of Annual average Solar radiation Maps (ASMs) derived from global DEMs. The framework consists of several phases, including data preprocessing, annual solar radiation estimation, ASM selection, data preparation for deep learning, model evaluation and selection, and model deployment.

Initially, the LiDAR DEMs with a spatial resolution of 0.5 m and the SRTM, ASTER, ALOS, and Copernicus DEMs with a spatial resolution of one arc-second are entered into the data preprocessing phase (Section 4.1). After coordinate system homogenization and resampling, the global DEMs with a spatial resolution of 30 m and the LiDAR DEM with a spatial resolution of 6 m are obtained in the UTM zone 31N/32N coordinate system. Subsequently, an annual solar radiation model, provided by the “Area Solar Radiation” tool in ArcGIS Pro 3.0, is employed to generate the maps of the annual average (year 2021) solar energy potential (ASMs) from the global and LiDAR DEMs (Section 4.2). In other words, at each location, the average solar energy potential during the hours of sunlight availability is calculated for each day of the year 2021.

In the ASM selection stage (Section 4.3), the objective is to select a more accurate ASM (obtained from the global DEMs) compared to the ASM derived from LiDAR. To achieve this, the ASM derived from LiDAR is first resampled to a spatial resolution of 30 m within the extent of the global DEMs. Using the 30 m ASM derived from LiDAR, the accuracy of ASMs obtained from the global DEMs is calculated. Subsequently, the most accurate ASM is selected for resolution enhancement and accuracy improvement.

In the data preparation for the deep learning stage (Section 4.4.1), the train and test regions are selected. Then, using the ASM derived from the LiDAR DEMs, train and test datasets are generated within the spatial extent of the train and test regions, with a target resolution of 6 m and an initial resolution of 30 m. Next, deep learning models are trained to improve the resolution of the LiDAR ASM. In the model evaluation and selection stage, the best-performing model (EDSR) is selected based on the performance of implemented models on the test data.

In the model deployment stage (Section 4.5), the trained model, by LiDAR data is employed to improve the resolution of ASM derived from the Copernicus DEM with a 30 m spatial resolution. The objective is to enhance the resolution of ASM by a factor of 5. In the final stage (Section 4.6), the accuracy of the ASM obtained from the Copernicus DEM with a 6 m spatial resolution, predicted by the selected super-resolution model, is calculated and evaluated. The evaluation involves comparing the enhanced ASMs with the ASM derived from the LiDAR DEMs. Further details of each of these stages will be explained in next sections.

### 4.1 Data preprocessing

The SRTM and ASTER DEMs encompassed the study area of the Netherlands with a total of 11 tiles. Each tile had dimensions of 1801 × 3601 pixels and a spatial resolution of 1 arc-second (approximately 30 m). In contrast, the ALOS and Copernicus DEMs were obtained as a single, contiguous tile that covers the entirety of the Netherlands study area. These two tiles were cropped to match the extent of the SRTM and ASTER DEM tiles.

**Figure 4:**
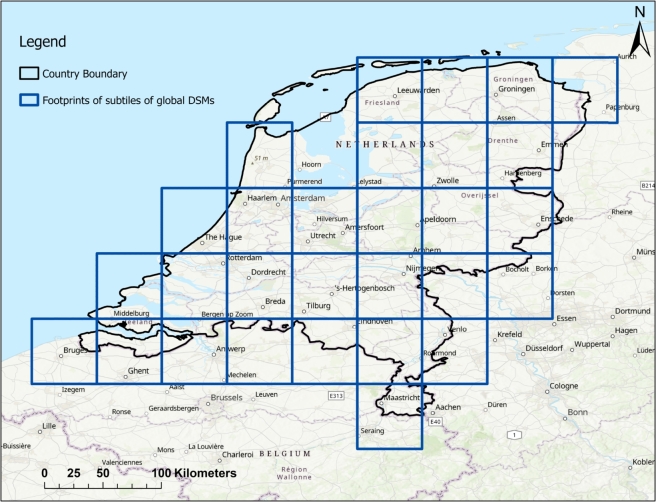
The extent of sub-tiles of global DEMs (SRTM, ASTER, ALOS, Copernicus) covering the study area.

Due to computational and hardware resource limitations, each of the DEM tiles was split into four equal parts, and sub-tiles outside the study area were removed. As a result, the number of sub-tiles for each DEM was reduced to 29 after the split and removal of those that did not cover the Netherlands. Fig. 4 depicts the extent of the sub-tiles covering the study area. Considering that the Netherlands is located in UTM Zones 31 and 32 in the northern hemisphere, the sub-tiles of the DEMs were projected to the UTM Zone 31N/32N horizontal reference coordinate system and EGM 1996 Geoid vertical reference. Subsequently, all sub-tiles of the SRTM, ASTER, ALOS, and Copernicus DEMs were resampled to match the spatial resolution of 30 m within the specific extent.

The number of LiDAR DEM tiles covering the Netherlands was 1121. Each tile had dimensions of 12500 × 10000 pixels, with a pixel size of 0.5 m. These tiles were also projected to the UTM Zone 31N/32N horizontal reference coordinate system and EGM 1996 Geoid vertical reference. Then they were resampled to a spatial resolution of 6 m. This preprocessing step was necessary to ensure consistency and compatibility among the tiles. Given the large volume of data and the high number of tiles, an automated mechanism was designed to apply all these preprocessing steps to the DEMs.

Since in computer vision, the resolution improvement scales for images are usually 3, 4 and finally 8 [45], [48], [46], in this research, the average of the lowest and the highest scales of super-resolution (i.e., 5 times) has been chosen for ASM resolution enhancement. Additionally ASM data are not similar to images in terms of color and texture, and choosing higher super-resolution scale may lead to decreasing the accuracy of ASM values.

### 4.2 Annual solar radiation estimation

Solar radiation, which originates from the sun, changes as it travels through the atmosphere. Topography and surface characteristics play a significant role in these variations. Solar radiation can be divided into direct, diffuse, and reflected. The sum of direct, diffuse, and reflected radiation is called global or total solar radiation. Direct radiation travels straight from the sun without obstruction, constituting the most significant portion of total radiation. Diffuse radiation is scattered by atmospheric components such as clouds and airborne particles and is the second-largest component of total radiation. The reflected radiation is reflected from surface features. Generally, it makes up only a tiny part of the total radiation, except in locations surrounded by highly reflective surfaces like snow cover. The solar radiation estimation model used in the Area Solar Radiation module of ArcGIS Pro 3.0 software does not consider reflected radiation in the calculation of total radiation. Therefore, total radiation is calculated as the sum of direct and diffuse radiation.

The Area Solar Radiation model estimates solar radiation in four stages:

1. Calculation of the upward-facing hemispherical view based on topography.

2. Overlaying the view on the sun map to estimate direct radiation.

3. Overlaying the view on the sky map to estimate diffuse radiation.

4. Thoroughly repeat the process for each location of interest to generate the radiation map.

Since topography and surface features can significantly influence solar radiation, a crucial component of calculating total radiation is generating an upward-facing hemispherical view for each location based on DSM. These hemispherical views are similar to upward-looking hemispherical photographs, capturing the entire sky from the ground. The amount of visible sky plays a vital role in determining the amount of radiation received at a given location. A viewshed is a raster representation of the entire sky, indicating which areas are visible and which are obstructed when viewed from a specific location [63]. The viewshed is calculated by searching in a specified number of directions around the location of interest and determining the maximum sky obstruction angle or horizon angle. For other directions not directly searched, horizon angles are interpolated. These horizon angles are then converted into a hemispherical coordinate system, representing a three-dimensional hemisphere of directions as a two-dimensional raster image. Each cell in this raster image is assigned a value indicating whether the direction of the sky is visible (white pixels) or obstructed (gray pixels). Viewsheds, along with sun maps (which provide information on the sun's position) and sky maps (which provide information on sky direction), are used to calculate direct, diffuse, and total (direct + diffuse) radiation at each location. This approach ensures that the model accurately captures the influence of topography and surface features on solar radiation.

Direct solar radiation originates from every direction in the sky and is calculated using the sun map within the viewshed. The sun map is a raster representation that shows the sun's path or apparent position throughout the hours of the day and across the days of the year. The sun map consists of distinct segments, each defined by the sun's position at specific intervals during the day (hours) and throughout the year (days or months). The sun's path is calculated based on the latitude of the study area and the time of year. The viewshed is overlaid onto the sun map to compute direct radiation. This allows for an accurate calculation of how much direct sunlight reaches a specific location, considering the sun's position throughout the day and year and accounting for any topographical obstructions that might block the sunlight.

As previously mentioned, diffuse radiation originates from all directions in the sky due to scattering by atmospheric components (such as clouds, particles, etc.). A sky map is created to calculate diffuse radiation for a specific location. The sky map provides a hemispherical view of the entire sky, which is divided into a series of sky segments. Diffuse radiation is calculated for each segment of the sky based on its direction, using the zenith and azimuth angles.

When calculating total radiation, the viewshed raster is overlaid with both the sun maps and the sky maps to determine the direct and diffuse radiation received from each direction in the sky. Finally, global radiation ($Global_{tot}$) is calculated as the sum of direct radiation ($Dir_{tot}$) and diffuse radiation ($Dif_{tot}$) for each pixel of DSM and is computed on a daily, monthly, and annual basis according to the following relationship [26], [63], [64]:

(1) $$Global_{tot}=Dir_{tot}+Dif_{tot}$$

In this study, annual average solar energy potential maps (ASMs) for the year of 2021 were generated automatically using the solar radiation estimation model within the Area Solar Radiation module. For each day of the year 2021, the daily average solar energy potential is calculated using the Area Solar Radiation model. Ultimately, the annual average of solar energy potential is obtained by averaging the daily solar energy potential values across all days of 2021. It should be noted that to compute the daily average solar energy potential at each location, the hourly average of solar energy potential is utilized during the hours when sunlight is available. The algorithm employed in the Area Solar Radiation model incorporates sky and sun maps and the effects of surrounding topographic shadows and cloud cover to calculate the solar energy potential received at each location. Table 2 represents the definition and configuration of the Area Solar Radiation module parameters in this study. To achieve a more accurate estimation of solar energy potential, the ASR model must be calibrated using ground measurements when available. According to Kausika et al. (2021) [43], these parameter values change annually; however, evaluations show that using default values for Transmittivity and Diffuse Proportion when precise measurements are unavailable results in minimal errors in estimating annual solar potential. Therefore, this study used default values to set up the ASR model for the Netherlands in 2021.

**Table 2:** Definitions and configurations of parameters in the *Area Solar Radiation Model*.

Parameter	Description	Value
Sky Size	The resolution of viewshed, solar, and sky maps.	200 (cells per side)
Day Interval	The time interval through the year (unit: days) that will be used to calculate sky sectors for the sun map.	1
Directions	The number of required computational directions related to the resolution of the input DEM.	16 (directions)
Diffuse Proportion	A portion of global normal radiation that is scattered, depending on atmospheric conditions.	0.3
Transmittivity	The ratio of energy reaching the Earth's surface to the energy received at the top of the atmosphere (extraterrestrial).	0.5

The maps were created for 1121 tiles of LiDAR DEMs with a spatial resolution of 6 m. Subsequently, these tiles were mosaicked within the extent of the global DEMs and resampled twice, once to a spatial resolution of 6 m and again to a spatial resolution of 30 m. This process was performed to align the ASMs derived from LiDAR DEMs with the global DEMs tiles. The resulting ASMs at resolutions of 6 m and 30 m were obtained for 29 sub-tiles depicted in Fig. 4 within the UTM Zone 31N/32N coordinate system.

The ASMs for the year of 2021 were also automatically generated for 29 sub-tiles of SRTM, ASTER, ALOS, and Copernicus DEMs with a spatial resolution of 30 m.

### 4.3 ASM selection

In order to evaluate the accuracy of the ASMs generated by global DEMs compared to the ASM obtained from LiDAR DEM, the evaluation metrics including Root Mean Square Error (RMSE), Mean Absolute Error (MAE), and Mean Absolute Percentage Error (MAPE) were calculated using the following eq. [15], [12], [31]:

(2) $$RMSE=\sqrt{\sum_{i=1}^{n}\frac{{\left({\overset{ˆ}{y}}_{i}-y_{i}\right)}^{2}}{n}}$$

(3) $$MAE=\frac{1}{n}\sum_{i=1}^{n}|{\overset{ˆ}{y}}_{i}-y_{i}|$$

(4) $$MAPE=\frac{100}{n}\sum_{i=0}^{n}|\frac{y_{i}-{\overset{ˆ}{y}}_{i}}{y_{i}}|$$

In the above equations, $y_{i}$ represents the pixel value in the ASM obtained from the LiDAR DEM (as the reference ASM), and ${\overset{ˆ}{y}}_{i}$ denotes the pixel value in the ASM obtained from a global DEM. *n* is the number of measurements (pixels).

RMSE measures the deviation or error between the ASM obtained from each of the global DEMs (SRTM, ALOS, ASTER, and Copernicus) and the ASM obtained from the LiDAR DEM. A smaller value of this metric indicates that the ASM derived from a global DEM has higher accuracy and less error compared to the ASM obtained from the LiDAR DEM.

Similarly, a smaller value of MAE illustrates that the absolute error between the ASM derived from a global DEM and the LiDAR-derived ASM is lower, also, indicating higher accuracy.

The MAPE expresses the percentage of relative error between the ASM of a global DEM and the LiDAR-derived one. This quantity ranges between 0 and 100 as a percentage. Therefore, as the value of this error approaches zero, it indicates a lower error and deviation between the ASMs derived from a global DEM and the LiDAR-derived DEM.

### 4.4 Deep learning-based super-resolution

#### 4.4.1 Data preparation for deep learning

**Figure 5:**
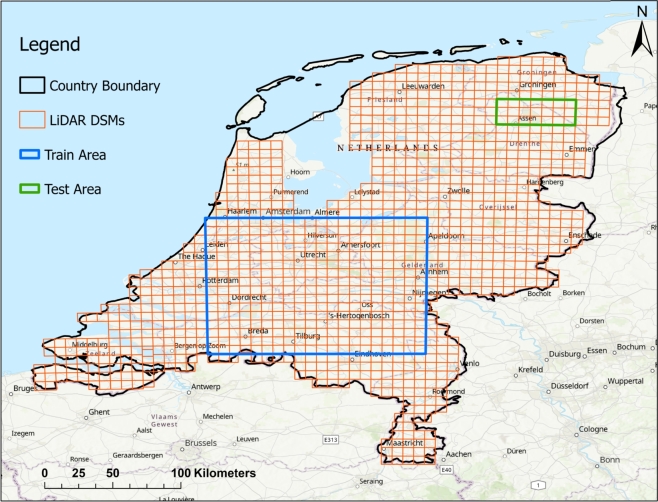
Extents of the train (blue) and test (green) regions defined for creating train and test data used in deep learning models.

In order to train deep learning models for improving resolution, it is necessary to define the training and testing datasets. For this purpose, two regions, namely train and test, were defined to create the training and testing data. The extents of the training and testing regions are represented by blue and green rectangles, respectively, as shown in Fig. 5. The train and test regions respectively consisted of 320 and 21 initial tiles of ASMs obtained from the LiDAR-derived DEMs with a spatial resolution of 6 m. To prepare the training and testing data, the initial tiles of LiDAR ASMs were first mosaicked within the train and test data extents. Then, patches with size of 100×100 pixels were generated as high resolution patches. Then, The generated patches were resampled to a spatial resolution of 30 m. Consequently, patches with size of 20×20 pixels were created as low resolution patches or inputs for the deep learning super-resolution model. The number of generated training and testing patches from the LiDAR-derived ASMs within the train and test data extents was 29,584 and 2,006 patches, respectively.

#### 4.4.2 Super-resolution models

The process of recovering high resolution (HR) images from one or multiple corresponding low resolution (LR) images is known as super-resolution (SR). Depending on the number of inputs LR images, SR can be classified into Single Image Super-Resolution (SISR) and Multi-Image Super-Resolution (MISR). SISR has received more attention due to its higher efficiency compared to MISR [86]. In this study, deep learning-based super-resolution models; Enhanced Deep Super-Resolution (EDSR), and U-Net-based super-resolution models with different backbones including ResNet18, ResNet34, ResNet50, and ResNet101, were utilized.

**Figure 6:**
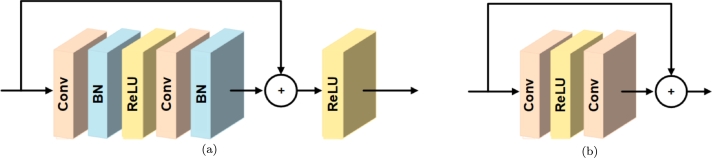
a) Typical ResNet network structure, b) ResNet network structure used in EDSR super-resolution model.

EDSR is a single-scale architecture that manages a specific scale of resolution improvement. This architecture utilizes the Residual Network (ResNet) as its backbone, which is widely employed in computer vision tasks [29], [44], [49]. Ledig et al. successfully employed ResNet in the Super-Resolution Residual Network (SRResNet) model [49]. Lim et al. by introducing modifications to the ResNet structure, introduced the EDSR super-resolution model, which outperformed the SRResNet model in terms of performance [50]. Fig. 6.a illustrates the main structure of the typical ResNet [29], while Fig. 6.b depicts the modified structure of the ResNet used in the EDSR super-resolution model. In the improved ResNet structure, the Batch Normalization (BN) layers have been removed compared to its original structure. This is because these layers normalize features within the range of zero to one, resulting in the reduction of range flexibility and the creation of compression. Additionally, features that fall outside the normalized range are also lost. Furthermore, BN layers consume memory resources similar to the preceding convolutional layers, so their removal can lead to memory savings on GPUs, particularly in resource-limited scenarios during the training process [50]. To improve the performance of convolutional networks like ResNet, it is necessary to increase the number of parameters [50]. One approach to increasing the number of parameters is by increasing the depth of the network. However, this leads to a more complex network that may not perform well under limited computational resources. Therefore, it is better to increase the number of feature filters in order to enhance the performance of a regular convolutional network. Nevertheless, increasing the number of feature filters beyond a certain limit can result in numerical instability during the training process, leading to oscillations in the loss values. To address this issue, a Residual Scaling with a constant factor of 0.1 can be applied. This means that after the last convolutional layers in the ResNet block structure, Residual Scaling layers are added [79].

**Figure 7:**
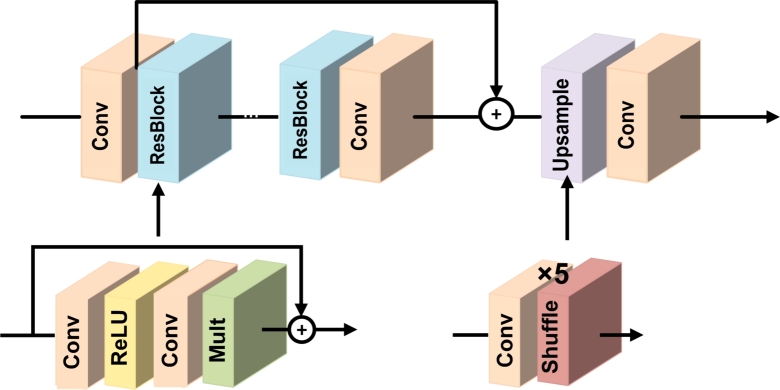
Structure of the EDSR single-scale super-resolution network used in this study.

The structure of the single-scale EDSR model used in this study is depicted in Fig. 7. According to the figure, the EDSR architecture consists of multiple identical residual blocks, where the low-resolution image (20 × 20-pixel patches from the ASM derived from LiDAR DEM with 30 m spatial resolution) is fed into the network. This input image passes through a convolutional layer to extract initial features. It is then fed into the core of the EDSR network, which is composed of multiple residual blocks.

Each residual block consists of several convolutional layers for feature extraction (Conv), a ReLU activation function, and residual connections to directly propagate low-frequency information from the input to the output of each residual block. The Mult layer (Mult) module in the ResBlocks enables EDSR to perform spatial resolution improvement at different scales. This module extracts features from multiple scales of the input image, allowing the network to generate high resolution outputs for different levels of spatial resolution enhancement. By incorporating the Mult module into the architecture, the EDSR network gains the ability to effectively capture and utilize multi-scale information, thus enhancing its capability to improve spatial resolution across various levels [51]. After passing through the residual blocks, the features are processed by upsampling layers to increase the spatial resolution. The shuffling layer in upsampling module is responsible for enhancing spatial resolution. This operation is performed using a convolutional layer with a specified number of filters to generate an output image with a larger size. Finally, the feature maps are processed by a convolutional layer to generate the high resolution output. By repeating the residual blocks and upsampling multiple times, the network can gradually learn more complex and precise features, leading to improved resolution. The number of filters used in the convolutional layer is equal to the product of the number of channels in the input image and the square of the scaling factor. Subsequently, the output image is produced with the desired increase in spatial resolution [51].

In this study, the EDSR employed a base model consisting of 16 ResNet blocks. In this model, ReLU activation layers were not used outside the residual blocks. Additionally, the number of feature maps for each convolutional layer was set to 32, and residual scaling layers were not employed [50]. Also the L1 norm was used as loss function in this network.

**Figure 8:**
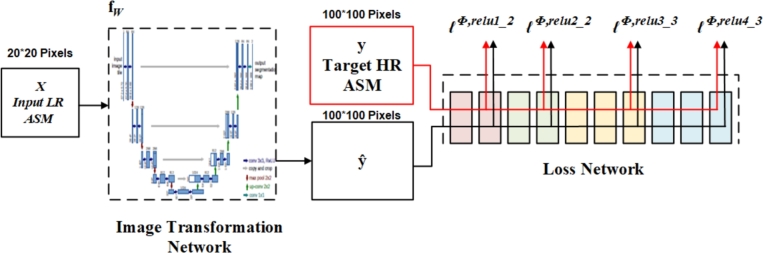
Structure of super-resolution network based on U-Net. The U-Net network is used as the Image Transform network and VGG-16 is used as the Loss network to calculate perceptual Loss.

In this research, in addition to the EDSR model, a U-Net-based super-resolution model was used to enhance the resolution of ASM maps. Fig. 8 illustrates the structure of this model. The network consists of two main parts. The first part of this network is the Image Transformation Network ($f_{w}$), and the second part is the Feature Loss Network *ϕ*, which defines different loss functions $\ell_{1}$, ..., $\ell_{k}$ (where k is the number of feature levels) for loss calculation. The U-Net model is considered as the Image Transformation Network, and VGG-16 is used as the Feature Loss Network.

In the first phase, the Image Transformation Network ($f_{w}$) takes the low resolution ASM patches as input images (x), performs transformations on them, and converts them into output images (high resolution ASM patches, $\overset{ˆ}{y}=f_{w}(x)$). In this research, U-Net with various backbone architectures, including ResNet 18, 34, 50, and 101, was used as the Image Transformation Network.

The U-Net was initially introduced for biomedical image segmentation [65]. Its architecture can be viewed as an encoder-decoder network. The first part of the U-Net architecture is typically a pre-trained classification network such as VGG or ResNet, where convolutional blocks followed by downsampling (maxpooling) operations are applied to extract features at different levels from the input image [65]. The second part of the U-Net architecture is the decoder, which semantically represents the learned distinctive features (low resolution) from the encoder onto the pixel space (higher resolution) to achieve semantic segmentation. The decoder consists of upsampling, concatenation, and subsequent convolution operations. In upsampling, the compressed feature map is restored to the original size of the input image, thereby expanding the feature dimensions. During upsampling in the network, feature maps with higher resolution from the encoder network are concatenated with the upsampled features to better learn representations through convolutional layers. Since upsampling is a sparse operation, a good representative of the initial stages is needed for better localization. Upsampling is also referred to as transposed convolution, upconvolution, or deconvolution. Existing methods for upsampling include nearest neighbor, bilinear interpolation, and transposed convolution. Additionally, the use of residual connections in U-Net networks is considered an attractive characteristic for image transformation networks, as in most cases, the output image needs to share a structural resemblance with the input image [29].

In the second phase, a Loss Network calculates the cost value $\ell_{i}$ (${\overset{ˆ}{y}}_{i}$, $y_{i}$), which represents the difference between the output image (${\overset{ˆ}{y}}_{i}$) and the target image ($y_{i}$). To better measure perceptual differences, pre-trained classification networks are used as the Loss Network [76], [53], [87]. In this research, VGG-16 was used as the Loss Network. VGG-16 is pre-trained on ImageNet data [75], [67], where the weights remain fixed during the training process. For the feature layers of this network at different levels, a loss is calculated. The model aims to ensure that each pixel of the generated image closely matches each pixel of the target image based on the pixel-wise loss. However, although these two images may appear similar in terms of perspective, they may have different values in each pixel, resulting in a blurry image. To improve this, Perceptual Loss is used, which combines per-pixel loss and feature loss from different layers of the Loss Network. It captures both the pixel-level differences and high-level feature representations extracted from a pre-trained CNN. Instead of encouraging the network to precisely match the pixels of the output image from the Image Transform Network ($\overset{ˆ}{y}=f_{w}(x)$) with the pixels of the target image (y), the network is encouraged to have similar feature representations calculated by the Loss Function *ϕ*. If $\phi_{j}$ is the activation function of layer j in the Loss Function *ϕ* for input image x, and j is a convolutional layer, then $\phi_{j}(x)$ will be a feature map with dimensions $C_{j}H_{j}W_{j}$. The loss of feature representation is the Euclidean distance (squared and normalized) between the feature representations [40]:

(5) $$\ell^{\phi ,j}(\overset{ˆ}{y},y)=(\frac{1}{C_{j}\times H_{j}\times W_{j}}){\left\|\phi_{j}(\overset{ˆ}{y})-\phi_{j}(y)\right\|}_{2}^{2}$$

In the overall process, a low resolution image is fed into the Image Transformation network, which predicts $\overset{ˆ}{y}$ as a high spatial resolution image. Then, the predicted images $\overset{ˆ}{y}$ and the ground truth images y are fed into the Loss Network, where the Perceptual Loss between the two images is computed.

In addition to Perceptual Loss, two other loss functions called Pixel Loss and Total Variation Loss have been utilized. These two loss functions are dependent on low-level pixel information. Pixel Loss is essentially the Euclidean distance (normalized) between the predicted image $\overset{ˆ}{y}$ and the ground truth image y. If the dimensions of the two images are equal to C × H × W, then Pixel Loss ($\ell_{pixel}(\overset{ˆ}{y},y)$) is computed as follows [40]:

(6) $$\ell_{pixel}(\overset{ˆ}{y},y)=(\frac{1}{C\times H\times W}){\left\|\overset{ˆ}{y}-y\right\|}_{2}^{2}$$

The Total Variation is a measure of the variation or changes in intensity across neighboring pixels in an image, and it promotes smoothness in the image. To encourage spatial smoothness in the output image, Total Variation Loss is utilized, which is calculated as follows [9]:

(7) $$\ell_{TV}(\overset{ˆ}{y},y)={\left\|y-\overset{ˆ}{y}\right\|}_{2}^{2}+\gamma {\left\|\nabla y\right\|}_{2}^{2},$$

where, *y* denotes the high resolution ground truth image, $\overset{ˆ}{y}$ represents the corresponding high resolution image generated by the super-resolution model, ∇*y* is the gradient of ground truth image which controls sharpness and smoothness of edges, and *γ* is a regularization parameter that balances the fidelity term and the penalty term in the equation. Increasing *γ* leads to the promotion of sharpness in the image generated by the super-resolution model.

The final high resolution image $\overset{ˆ}{y}$ is produced by minimizing the following total loss:

(8) $$\overset{ˆ}{y}=arg\min_{y}\{\lambda_{1}\ell^{\phi ,j}(\overset{ˆ}{y},y)+\lambda_{2}\ell_{pixel}(\overset{ˆ}{y},y)+\lambda_{3}\ell_{TV}(\overset{ˆ}{y},y)\},$$

where $\lambda_{1}$, $\lambda_{2}$, and $\lambda_{3}$ are scalars in order to weigh each Loss.

#### 4.4.3 Model setup

In this research, the EDSR super-resolution model with 16 residual blocks in the backbone was employed to enhance the resolution of solar energy maps. Additionally, a U-Net-based super-resolution model with different backbones, namely ResNet 18, 34, 50, 101, and a Loss Network using VGG-16, was developed for calculating the Perceptual Loss. Table 3 provides detailed specifications of the hyperparameters tuned for these models. All models were trained with a batch size of 8 over 20 epochs.

**Table 3:** Setting hyperparameters for the super-resolution models.

Model	Backbone	Number of Epochs	Batch Size	Learning Rate	Loss Function
EDSR	ResNet 34	20	8	1 × 10^−4^	L1
U-Net based	ResNet 18	20	8	4 × 10^−4^	eq. (8)
U-Net based	ResNet 34	20	8	1.5 × 10^−4^	eq. (8)
U-Net based	ResNet 50	20	8	1 × 10^−4^	eq. (8)
U-Net based	ResNet 101	20	8	3 × 10^−4^	eq. (8)

#### 4.4.4 Performance evaluation

In order to evaluate the accuracy and select the super-resolution model with the best performance, relative RMSE (rRMSE), relative MAE (rMAE), relative Mean Squared Error (rMSE), Structural Similarity Index Measurement (SSIM) and Peak Signal-to-Noise Ratio (PSNR) metrics have been used as evaluation quantities for validation and accuracy assessment. A smaller calculated rRMSE indicates that the super-resolution model has higher accuracy and stability. Eq. (9) to 11 illustrates the formulation of rRMSE, rMSE and rMAE.

(9) $$rRMSE=\frac{1}{m}\sqrt{\sum_{i=1}^{m}\frac{\sum_{p=1}^{l}{\left(y_{pi}-{\overset{ˆ}{y}}_{pi}\right)}^{2}}{l\times {\left(mean(y_{pi})\right)}^{2}}}$$

(10) $$rMSE=\frac{1}{m}\sum_{i=1}^{m}\frac{\sum_{p=1}^{l}{\left(y_{pi}-{\overset{ˆ}{y}}_{pi}\right)}^{2}}{l\times {\left(mean(y_{pi})\right)}^{2}}$$

(11) $$rMAE=\frac{1}{m}\sum_{i=1}^{m}\frac{\sum_{p=1}^{l}|y_{pi}-{\overset{ˆ}{y}}_{pi}|}{l\times mean(y_{pi})}$$

In the given equations; m is the number of image patches of the ASM, *l* represents the number of pixels in each image patch, $y_{pi}$ denotes the value of pixel p in the image patch i of the LiDAR0-derived ASM (reference ASM), and ${\overset{ˆ}{y}}_{pi}$ represents the value of pixel p in the image patch i generated by the super-resolution model.

The rMSE and rRMSE measure the deviation or error between the high resolution patches generated by the super-resolution model and the reference high resolution patches. They quantify the difference between these two sets of image patches, considering a spatial resolution of 6 m. A smaller value of these errors indicates more similarity between patches generated by the super-resolution model and the reference high resolution patches.

A smaller rMAE value indicates a lower overall error between the high resolution patches generated by the super-resolution model and the reference high resolution patches.

SSIM is a metric that quantifies the structural similarity between the high resolution LiDAR ASM patches produced by the super-resolution model and the reference high resolution LiDAR ASM patches. A higher SSIM value indicates a greater similarity between the generated patch by the super-resolution model and the reference patches. SSIM is calculated using the following eq.:

(12) $$SSIM(I,\overset{ˆ}{I})={\left(\frac{2\times \mu_{I}\times \mu_{\overset{ˆ}{I}}+C_{1}}{\mu_{I}^{2}+\mu_{\overset{ˆ}{I}}^{2}+C_{1}}\right)}^{\alpha }.{\left(\frac{2\times \sigma_{I}\times \sigma_{\overset{ˆ}{I}}+C_{2}}{\sigma_{I}^{2}+\sigma_{\overset{ˆ}{I}}^{2}+C_{2}}\right)}^{\beta }.{\left(\frac{\sigma_{I\overset{ˆ}{I}}+C_{1}}{\sigma_{I\overset{ˆ}{I}}+C_{1}}\right)}^{\gamma },$$

where, $\sigma_{I}$ and $\mu_{I}$ denote the mean and standard deviation of the reference high resolution LiDAR ASM patches (I), respectively. $\sigma_{\overset{ˆ}{I}}$ and $\mu_{\overset{ˆ}{I}}$ represent the mean and standard deviation of the high resolution LiDAR ASM patches generated by the super-resolution model ($\overset{ˆ}{I}$), respectively. $\sigma_{I\overset{ˆ}{I}}$ is the cross-covariance between the reference high resolution LiDAR ASM patches and the high resolution LiDAR ASM patches generated by the super-resolution model. *α*, *β*, and *γ* are constant parameters, usually set to 1 for simplicity. $C_{1}$, $C_{2}$, and $C_{3}$ are small positive constants used to prevent computational instability when the denominator of the fraction is small [71].

PSNR is a measure of the ratio between the peak signal power and the noise power in two images, expressed in decibels. It is used to assess the quality of the high resolution LiDAR ASM patches generated by the super-resolution model relative to the quality of the reference high resolution LiDAR ASM patches. The PSNR is calculated as follows [20]:

(13) $$PSNR=20\log_{10}(\frac{L}{RMSE})=10\log_{10}(\frac{L^{2}}{MSE}),$$

where L is most of the changes in input light (range of value changes in the image). For example, in an 8 bit image, the value of L is equal to $2^{8}=256$.

### 4.5 Model deployment

After evaluating the accuracy of deep learning-based super-resolution models, the model with the best performance was selected as the preferred super-resolution model. As will be demonstrated in Section 5.2, the EDSR model will achieve the highest accuracy in improving the resolution of LiDAR data from 30 m to 6 m. Subsequently, the ASM generated by the global DEM with the highest accuracy was chosen as the final ASM (determined in the ASM Selection process) for resolution enhancement by the selected deep learning model. As will be shown in Section 5.1, the ASM derived from the Copernicus DEM with a spatial resolution of 30 m will exhibit higher accuracy compared to other ASMs and will be selected as the preferred ASM. To enhance the resolution of the selected ASM, the data needed to be prepared for input into the model. Therefore, patches with size of 20 × 20 pixels were generated from each selected ASM sub-tile. The total number of patches generated at this stage was 149,609 patches with size of 20 × 20 with a resolution of 30 m, which were used as input to the super-resolution model. The corresponding high resolution patches with size of 100 × 100 with a resolution of 6 m were predicted by the model. Then, the predicted high resolution ASM patches of the Copernicus DEM were mosaicked, resulting in an integrated 6 m resolution ASM map for the entire study area. In this way, the 30 m ASM map of the Netherlands derived from the Copernicus DEM was enhanced to 6 m using the developed deep learning-based super-resolution model.

### 4.6 Accuracy assessment of generated high-resolution ASM by transfer learning

To assess the generalization and transferability of the selected deep learning model (EDSR) in enhancing the resolution of the chosen ASM (Copernicus), evaluation metrics such as RMSE, MAE, and MAPE were utilized (Section 4.3).

## 5 Result

### 5.1 Accuracy assessment of ASMs derived from global DEMs

**Figure 9:**
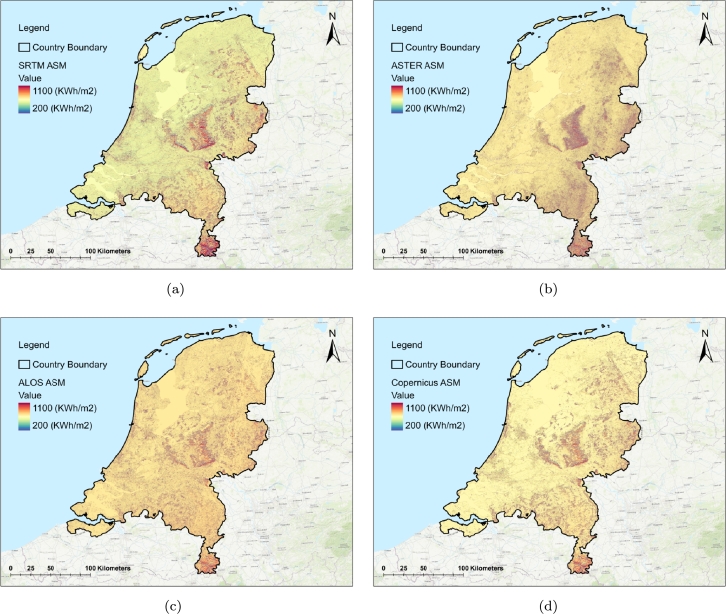
The ASMs derived from (a) SRTM DEM, (b) ASTER DEM, (c) ALOS DEM, (d) Copernicus DEM.

**Figure A.1:**
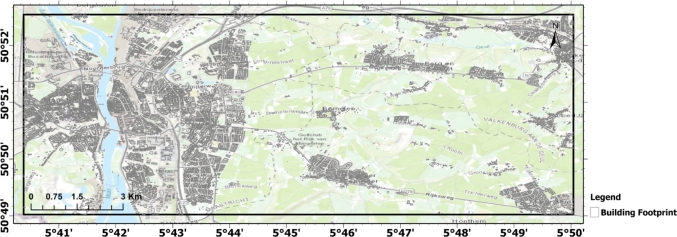
Visualization of the extent of an exemplary selected area in Limburg Province, including building footprints.

**Figure A.2:**
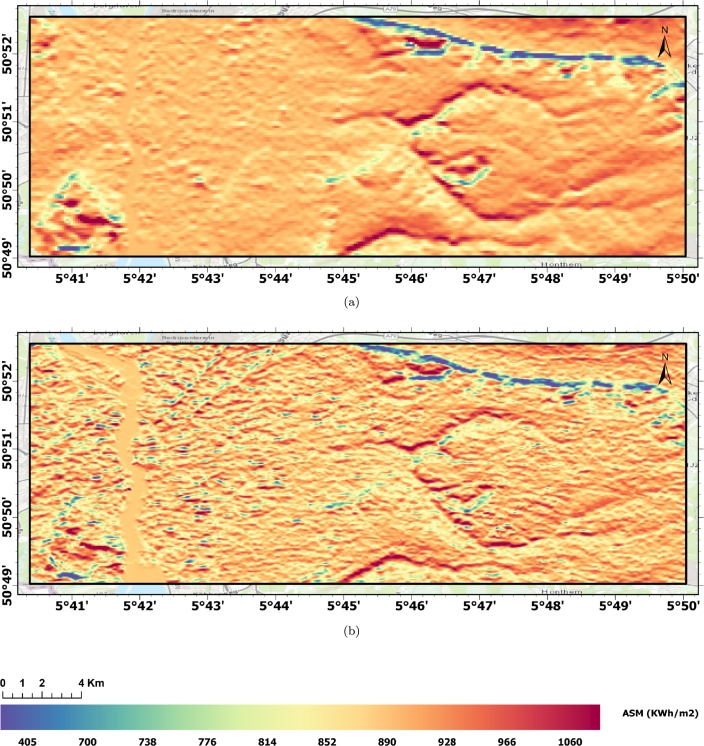

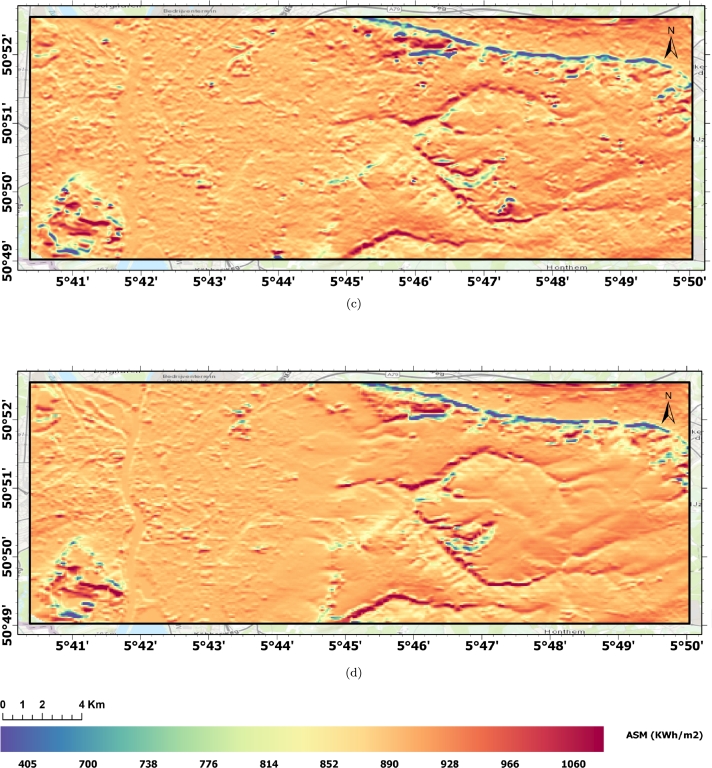
ASMs derived from (a) SRTM DSM, (b) ASTER DSM, (c) ALOS DSM, and (d) Copernicus DSM for the selected area in Limburg Province.

**Figure A.3:**
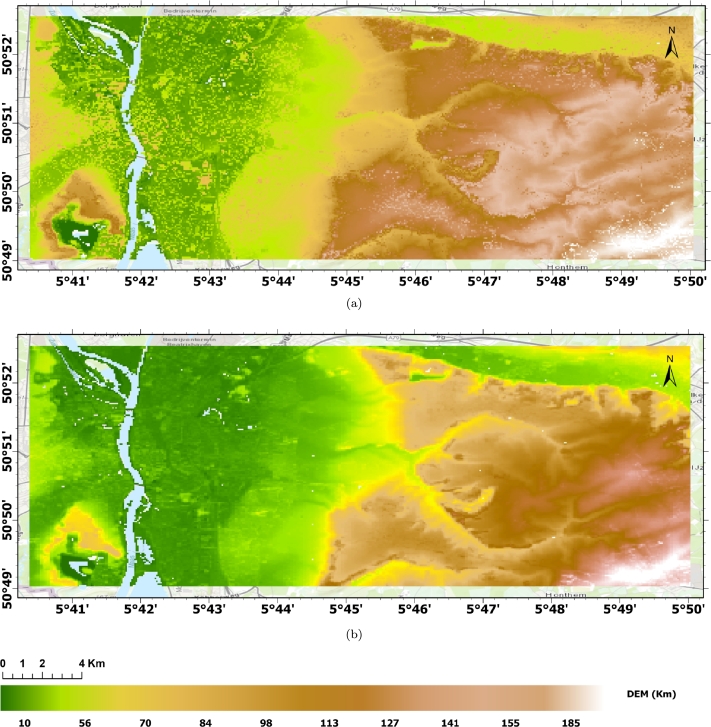
Topographic visualization of the selected area in Limburg Province using the 30 m LiDAR DSM (a) and the 30 m Copernicus DSM (b).

Fig. 9, displays the ASMs derived from global DEMs (SRTM, ASTER, ALOS, Copernicus). While all four maps exhibit a similar pattern, they differ in the level of detail. The highest solar energy potential is observed in the central and southern parts of the Netherlands, where topographical features like hills are present (see also Fig. 17 representing 30 m LiDAR and Copernicus DEMs). Conversely, in the Rhine, Meuse, and Scheldt river deltas, the solar energy potential decreases. Fig. A.1 displays the extent of the exemplary selected area (located in Limburg province, the southern Netherlands) to visualize close-up maps of ASMs derived from different DEMs. Correspondingly, Fig. A.2 provides a close-up look at ASMs, and Fig. A.3 shows the topographic visualization of the same area using 30 m LiDAR and Copernicus DEMs.

The left side of the map represents an urban area, while the right side depicts a non-urban area ranging from flatlands to complex topographies. The ASM map shows that the solar potential values increase in areas with complex topographies. In contrast, solar potential values decrease significantly in flat regions and exhibit more uniform variations. In non-urban areas, the ASM derived from Copernicus displays solar potential variations (due to topographic changes), perfectly with less noise than other ASMs. In urban areas, the Copernicus-derived ASM also has a higher quality than those from other DEMs, accurately depicting the potential solar energy changes relative to elevations (see Fig. A.3), especially at the edges of buildings, and other urban infrastructures.

Table 4 presents the accuracy of ASMs generated by global DEMs (SRTM, ASTER, ALOS, Copernicus) compared to the ASM derived from the LiDAR DEMs within the entire study area of the Netherlands. This accuracy assessment was also conducted for four selected cities: Amsterdam, Rotterdam, Eindhoven, and Assen. The SRTM and Copernicus-derived ASMs exhibited a closer resemblance (lower error) to the LiDAR-derived ASM in comparison to ASTER and ALOS.

**Table 4:** Accuracy assessment of ASMs obtained from global DEMs with a spatial resolution of 30 m in the entire study area as well as selected urban areas of the Netherlands.

Area	DEM	RMSE($\frac{kWh}{m^{2}}$)	MAE($\frac{kWh}{m^{2}}$)	MAPE(%)
Whole Area of the Netherlands	SRTM (30 m)	**107.5**	51.1	28.3
	ASTER (30 m)	108.7	53.4	28.4
	ALOS (30 m)	109.3	52.7	28.6
	Copernicus (30 m)	108.0	**49.7**	**28.2**

Selected Urban Areas	SRTM (30 m)	**166.2**	103.9	18.9
	ASTER (30 m)	168.4	105.0	19.1
	ALOS (30 m)	168.4	104.9	19.2
	Copernicus (30 m)	166.9	**103.5**	**18.9**

In the entire study area of the Netherlands, the RMSE value for the SRTM-derived ASM is 107.498 $\frac{kWh}{m^{2}}$, which is slightly lower than the RMSE value for the Copernicus-derived ASM (107.974 $\frac{kWh}{m^{2}}$). However, the MAPE value for the Copernicus-derived ASM in the entire study area is 0.072% lower than the MAPE value for the SRTM-derived one. Furthermore, The MAE value for the Copernicus-derived ASM in the entire study area of the Netherlands is 1.354 $\frac{kWh}{m^{2}}$ smaller than the MAE value for the SRTM-derived ASM. Therefore, in the overall study area, the Copernicus-derived ASM exhibits slightly higher accuracy compared to the SRTM-derived ASM.

In the urban areas of the study region in the Netherlands, the RMSE value for the ASM derived from SRTM is 166.248$\frac{kWh}{m^{2}}$, which is slightly lower than the RMSE value for the ASM derived from Copernicus (166.887 $\frac{kWh}{m^{2}}$). However, The MAE value for the Copernicus-derived ASM in the urban areas of the Netherlands is 0.38 $\frac{kWh}{m^{2}}$ smaller than the MAE value for the SRTM-derived ASM. The MAPE value for the Copernicus-derived ASM in the urban areas of the study region is 0.004% lower than the MAPE value for the SRTM-derived ASM. Therefore, in urban areas and also in the entire study area of the Netherlands, the Copernicus-derived ASM is slightly more accurate compared to the SRTM-derived ASM.

Both the Copernicus and SRTM DEMs have a radar nature, which means they inherently possess radar induced errors and outliers, especially in complex topographies. However, in the third version of the SRTM DEM, it is void-filled using elevation data from ASTER, GDEM2, USGS GMTED 2010, and USGS NED. This integration reduces large errors and outliers, resulting in a lower RMSE for the SRTM-derived ASM compared to the Copernicus-derived ASM.

Considering that the Copernicus DEM was produced more recently (2011-2015) compared to the SRTM DEM (2000-2015), as well as the production year of the LiDAR DEM (2014-2019) and the year of estimating ASM (2021), the Copernicus-derived ASM was selected among all of them for further processing. Totally, based on the previous evaluations, the Copernicus DEM is the most accurate up-to-date DEM [54]. Thus, the derived ASM provides a better estimation of solar potential. Last but not least, in urban areas and the entire area of the Netherlands, the Copernicus DEM-derived ASM exhibits slightly higher accuracy. Therefore, the Copernicus-derived ASM provides the potential of solar energy more accurately.

### 5.2 Comparison of super-resolution models

The U-Net-based super-resolution models with ResNet 18, ResNet 34, ResNet 50, and ResNet 101 backbones, along with the EDSR super-resolution model, were trained on paired LR (30 m) and HR (6 m) patches of ASMs derived from the LiDAR DEM. The train process consisted of 20 epochs with a batch size of 8, and 20% of the train data was allocated for validation purposes.

**Figure 10:**
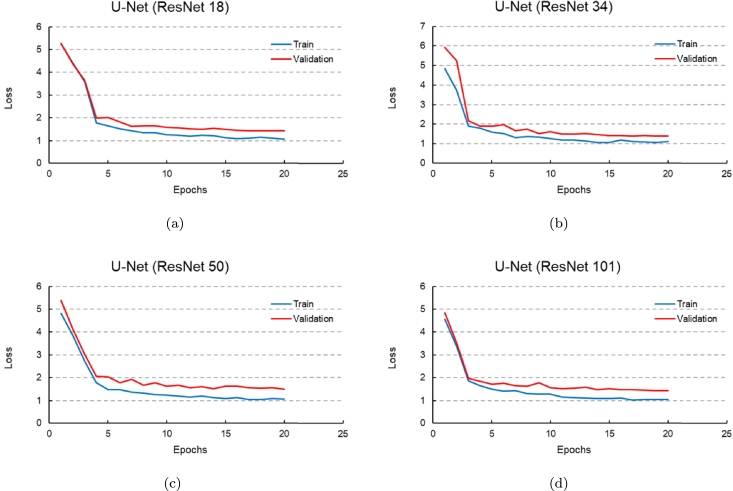
Train and validation loss plots; (a) U-Net with ResNet 18, (b) U-Net with ResNet 34, (c) U-Net with ResNet 50, (d) U-Net with ResNet 101.

Fig. 10 displays the loss plots of the U-Net-based super-resolution models with four different backbones (ResNet18, 34, 50, and 101) for both the train and validation data. Total loss (eq. (8)) was utilized as the loss function in these plots. In the U-Net-based super-resolution models with ResNet 50 and 101, increasing the number of epochs resulted in a reduction in the train loss. However, the validation loss increased compared to the U-Net-based super-resolution models with ResNet 18 and 34. This indicates that some degree of overfitting has occurred. In other words, as the number of ResNet blocks increases, the U-Net-based super-resolution model becomes more complex. With the same number of epochs, increasing the number of ResNet blocks (such as in ResNet 50 and 101) leads to the issue of overfitting. This means that the model fits well to the train data, resulting in a decrease in the train loss, but it struggles to generalize well to new data, such as the validation data, when performing super-resolution operations. Consequently, the validation loss increases.

**Figure 11:**
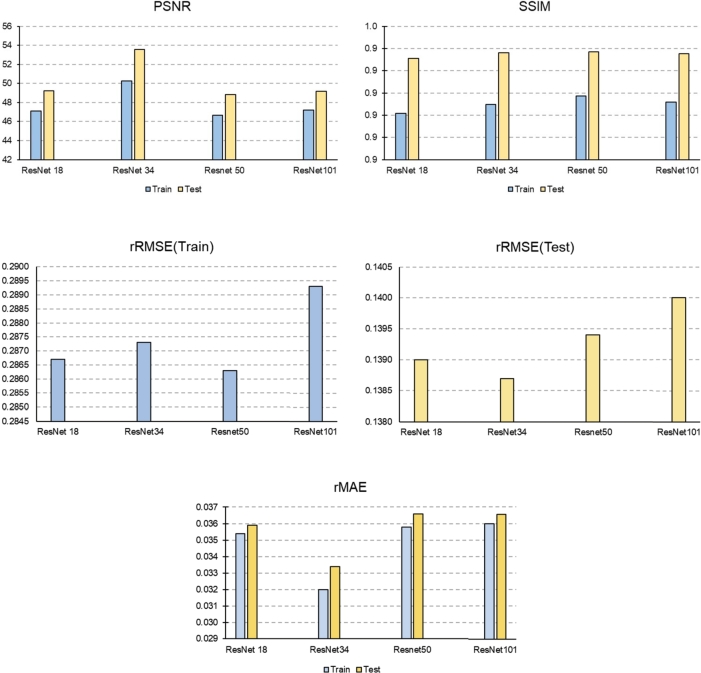
Comparison of train and test accuracy of U-Net-based super-resolution models with 5 different backbones based on PSNR, rRMSE, rMAE, and SSIM.

In Fig. 11, the plots of rRMSE, rMAE, PSNR, and SSIM values for U-Net-based super-resolution models with different backbones in the train and test modes are presented. In the train mode, the U-Net-based super-resolution model with a ResNet 50 achieved the lowest rRMSE value, while in the test mode, the ResNet 34 backbone yielded the lowest rRMSE value. Based on the rMAE parameter, the U-Net-based super-resolution model with a ResNet 34 demonstrated the best performance in both the train and test modes.

PSNR is a measure of the ratio between the maximum possible power of a signal and the power of corrupting noise. Thus, a higher PSNR value indicates the lower level of noise in the generated patches, better performance of the super-resolution model. In both the train and test modes, the U-Net-based super-resolution model with a ResNet 34 had the highest PSNR value.

SSIM is a measurement of the structural similarity index between digital images. It provides a method for measuring the similarity between the reference high resolution image and the high resolution image generated by the super-resolution model. The closer this value to one, implies more similarity between reference and generated images. In the high-resolution reference image, there is a correlation between the value of each pixel (representing the annual average solar energy potential) and its neighboring pixels. This correlation is expected to be present in the high resolution image generated by the super-resolution model. In other words, structural similarity implies that the correlation between each pixel and its neighbors in the model-generated high resolution image should resemble that in the reference high resolution image. The U-Net-based super-resolution model with a ResNet 50 achieved the highest SSIM value in both the train and test modes. However, the difference in the SSIM values among the U-Net-based super-resolution models with different backbones was at most 0.004 in the train mode and 0.001 in the test mode.

In conclusion, based on the loss plots of the train and validation data, as well as the plots of the rRMSE, rMAE, PSNR, and SSIM metrics in the train and test modes for different backbones, it can be inferred that the U-Net-based super-resolution model with a ResNet 34 produces high resolution images with lower error and higher structural similarity compared to other backbones. Additionally, the images produced by the U-Net-based super-resolution model with a ResNet 34 exhibit less noise and a closer resemblance to the reference high resolution images.

Table 5 compares the performance of the U-Net-based (ResNet 34) super-resolution model to the EDSR super-resolution model. In the train mode with 20 epochs, the U-Net-based (ResNet 34) super-resolution model achieved the lowest rRMSE, rMSE and rMAE values. Furthermore, The U-Net-based (ResNet 34) super-resolution model achieved the highest SSIM values. While, the EDSR super-resolution model had the highest PSNR value in the train mode. Generally, the U-Net-based (ResNet 34) model outperforms the EDSR model in the train mode with 20 epochs. In the test mode, the EDSR model achieved the lowest values for rRMSE, rMSE, and rMAE. Additionally, the EDSR model had the highest SSIM value, while the U-Net-based (ResNet 34) model achieved the highest PSNR value. In other words, the EDSR model performed better in enhancing the spatial resolution of image patches with low spatial resolution, which were unseen during its train process, compared to the U-Net-based (ResNet 34) model. Nevertheless, according to the PSNR parameter, the EDSR model introduces more noise in the generated patches compared to the U-Net-based (ResNet 34) model. Considering the SSIM values in the test mode, there is a higher structural similarity between the generated patches by the EDSR model and references compared to the U-Net-based (ResNet 34) model.

**Table 5:** Comparing the train and test accuracy of EDSR and U-Net based super-resolution models with the best performance (with ResNet 34 backbone).

Models	Train					Test				
	rRMSE	rMSE	rMAE	SSIM	PSNR (dB)	rRMSE	rMSE	rMAE	SSIM	PSNR (dB)
U-Net-based	0.2873	0.0825	**0.0320**	**0.9324**	50.2503	0.1387	0.0192	0.0334	0.9441	**53.5901**
EDSR	**0.2851**	**0.0813**	0.0365	0.9273	**50.872**	**0.1331**	**0.0177**	**0.0283**	**0.9508**	51.4515

**Figure 12:**
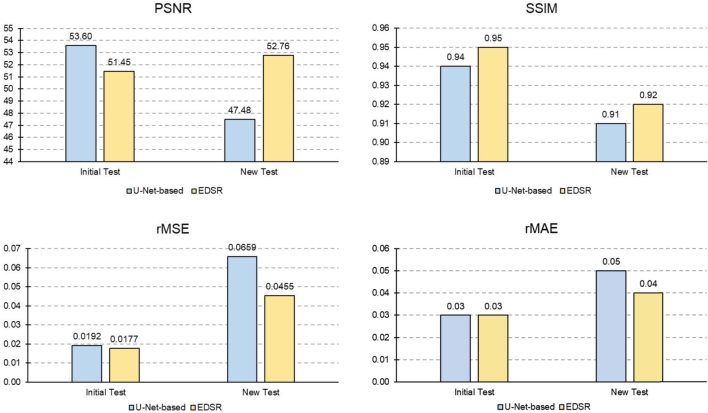
Generalizability assessment of the U-Net-based super-resolution model (ResNet 34) and the EDSR model in new test regions compared to the initial test area based on PSNR, rMSE, rMAE, and SSIM.

Furthermore, to assess the generalizability of the EDSR and U-Net-based (ResNet34) models in enhancing the spatial resolution of LiDAR ASMs, we evaluated their performance in new test regions with different geographical characteristics from the initial test and training areas. Fig. 12 compares these models' performance in the new test regions versus the initial test region.

As illustrated in Fig. 12, while the rMSE and rMAE metrics for the super-resolution models show an increase in the new test regions, and SSIM and PSNR parameters reflect a slight decline in the quality of the generated ASMs, these changes do not dramatically impact the overall performance of the models. Notably, the reduction in accuracy is less pronounced for the EDSR model than for the U-Net-based model. The EDSR model exhibits greater generalizability and is less sensitive to geographical variations than the U-Net-based model. Additionally, one advantage of deep learning-based super-resolution models is their ability to be fine-tuned with a limited amount of training data from new regions, which helps maintain performance accuracy across different locations.

**Figure 13:**
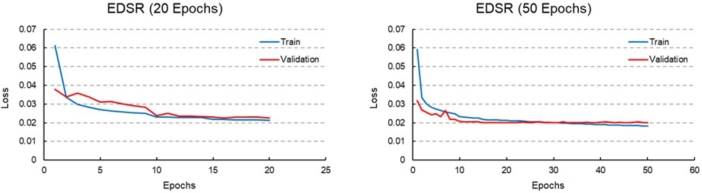
Loss plots of train and validation of EDSR super-resolution network with 20 and 50 epochs.

Therefore, it can be concluded that the EDSR model performs better in improving the spatial resolution of the ASMs derived from the LiDAR DEM compared to the U-Net-based (ResNet 34) model. Based on the loss reduction trends observed for both train and validation data (Fig. 13), it is expected that increasing the number of train epochs of EDSR model will lead to better accuracy in the spatial resolution enhancement process.

In Fig. 13, the loss plots for the EDSR model with 20 and 50 train epochs are displayed. The loss function used in this model is L1 loss. As the number of train epochs increased, the train and validation losses decreased. Therefore, in the following steps, the EDSR model which had been trained for 50 epochs was selected as the preferred model to improve the spatial resolution of low spatial resolution image patches of the ASM derived from Copernicus DEM.

**Figure 14:**
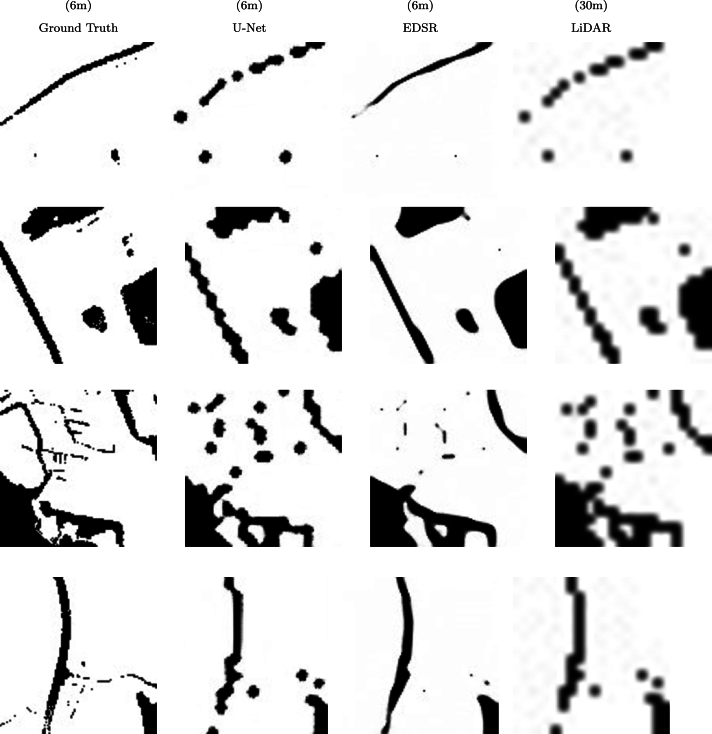
Exemplary patches of LiDAR-derived ASM at a spatial resolution of 6 m (ground truth) and 30 m along with patches produced by EDSR and U-Net-based super-resolution models. Note that for better comparison, the binary visualization of ASM patches has been utilized.

In Fig. 14, several patches of the LiDAR-derived ASM at spatial resolutions of 6 m and 30 m are displayed. Correspondingly, patches with a spatial resolution of 6 m obtained through the resolution enhancement by the U-Net based super-resolution model and the EDSR model are also shown. As depicted in Fig. 14, both super-resolution models have been able to improve the resolution of low resolution LiDAR-derived patches. The U-Net based super-resolution model has recovered the details in a coarse manner during the super-resolution process, while the EDSR super-resolution model has attempted to retrieve the details with a similar finesse to those present in the high-resolution reference patch. Furthermore, in terms of edge recovery, the U-Net (ResNet 34) based super-resolution model has produced sharp and jagged edges, whereas the EDSR super-resolution model has resulted in smoother edges, similar to the reference data.

### 5.3 Accuracy assessment and generalizability of EDSR model for super-resolution of Copernicus-derived ASM

After training deep learning models and selecting the best model, the trained model is applied to enhance the resolution of the ASM obtained from the Copernicus DEM with a spatial resolution of 30 m. Initially, these maps are divided into 20 × 20 patches and fed into the EDSR super-resolution model, resulting in resolution enhanced patches with a spatial resolution of 6 meters (100 × 100 size). By mosaicking these patches within the extent of the study area, an ASM with a 6 m spatial resolution is generated. Subsequently, the accuracy of the created 6-meter map by the EDSR model is evaluated against the LiDAR reference map in various land use categories, such as urban areas, non-urban areas, and highway margins. Table 6 presents the accuracy of the ASM obtained from the Copernicus DEM with a 30 m (original) and 6 m (predicted by the EDSR super-resolution model) spatial resolution compared to the LiDAR-derived ASM in the entire study area of the Netherlands.

**Table 6:** Accuracy evaluation of enhanced ASM (6 m) and the original ASM (30 m) derived from Copernicus respective to LiDAR-derived ASM.

Land Use	ASM	RMSE($\frac{kWh}{m^{2}}$)	MAE($\frac{kWh}{m^{2}}$)	MAPE (%)
Whole Area of the Netherlands	Copernicus 6 m	106.53	49.77	28.30
	Copernicus 30 m	107.97	49.84	28.24

Urban Area	Copernicus 6 m	**164.80**	**101.32**	**16.32**
	Copernicus 30 m	166.89	103.49	18.93

Non-Urban Area	Copernicus 6 m	**105.50**	**49.06**	28.39
	Copernicus 30 m	107.97	49.84	**28.37**

Highways	Copernicus 6 m	**111.18**	**57.28**	**22.83**
	Copernicus 30 m	112.83	57.92	35.45

In urban, and non-urban areas and highway margins, and also the entire area of the Netherlands, the generated ASM derived from the Copernicus DEM at a spatial resolution of 6 m shows a lower RMSE compared to the map with a spatial resolution of 30 m. In other words, the enhanced ASM by EDSR exhibits lower error and deviation compared to the original ASM with a 30 m spatial resolution. Furthermore, the MAE of the produced 6 m ASM is lower than the 30 m resolution in all the mentioned areas. The MAPE of the enhanced ASM is also better than the 30 m Copernicus-derived ASM in urban areas and highway surroundings. However, this metric has a slightly increased in non-urban areas as well as the whole area of the Netherlands. Overall, the EDSR super-resolution model has not only improved the resolution of the ASM derived from the original Copernicus DEM but also increased the accuracy of solar energy estimation, particularly in urban areas and highway margins. The achieved results also indicate the high accuracy, stability, and generalization capability of the EDSR super-resolution model.

**Figure 15:**
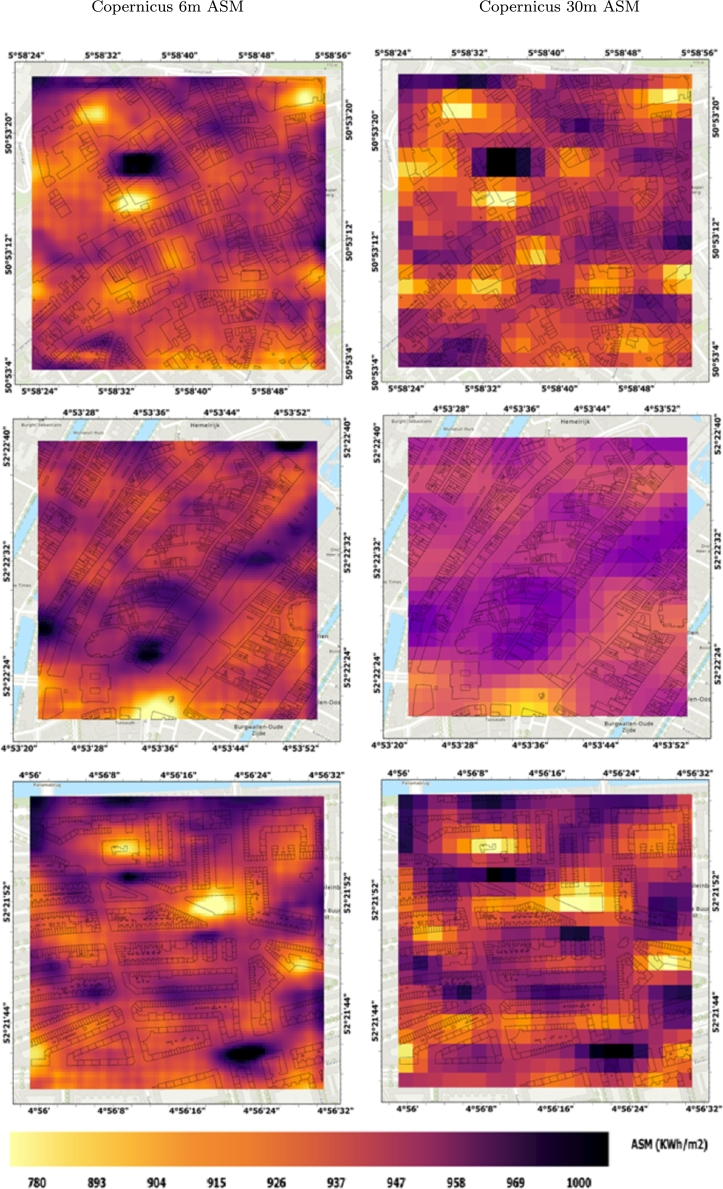
Exemplary patches of the enhanced Copernicus-derived ASM with a 6 m spatial resolution compared to the original 30 m Copernicus-derived ASM in some selected urban areas of the Netherlands.

Fig. 15 displays the enhanced Copernicus-derived ASM, processed using EDSR, alongside the ASM obtained from the original Copernicus DEM at a 30-meter resolution in selected urban areas of the Netherlands. As discussed earlier, the enhanced ASM provides more detail and is more suitable than the 30-meter resolution for rooftop solar radiation estimation.

**Figure 16:**
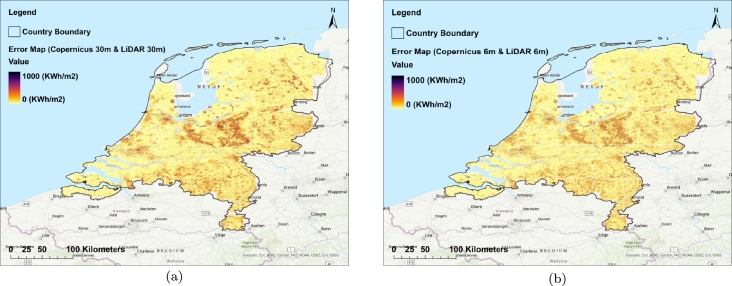
Absolute error maps; (a) the 30 m Copernicus-derived ASM respective to LiDAR reference map (b) the 6 m produced ASM by the EDSR model respective to LiDAR.

**Figure 17:**
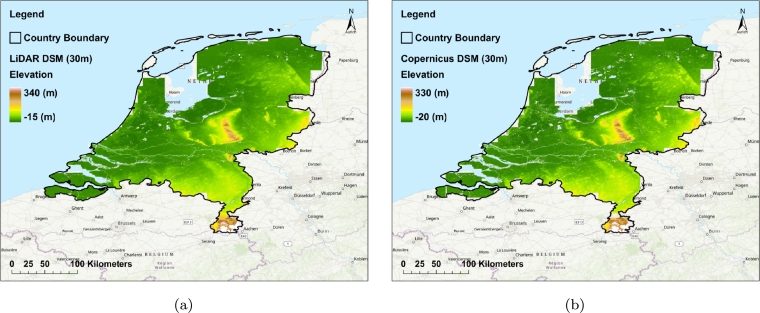
Topographic visualization of the Netherlands using the 30 m LiDAR DSM (a) and the 30 m Copernicus DEM (b).

**Figure A.4:**
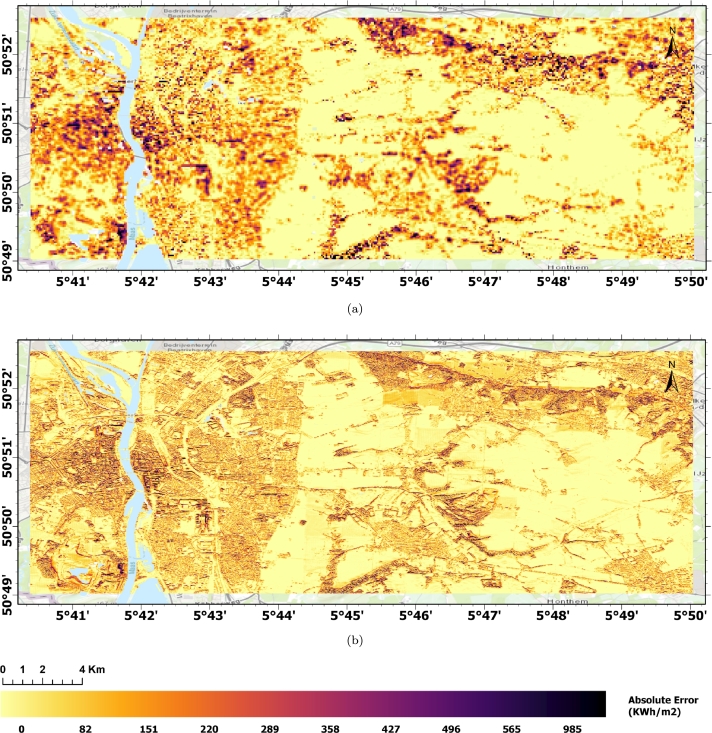
Absolute error maps of the selected area in Limburg Province: (a) 30 m Copernicus-derived ASM compared to the LiDAR reference map, and (b) 6 m ASM produced by the EDSR model compared to the LiDAR reference map.

In Fig. 16, the maps of absolute errors for the ASM derived from the Copernicus DEM (30 m) (a) as well as 6 m enhanced resolution by EDSR (b) respective to the ASM of LiDAR are displayed. Also, Fig. 17 displays the map of the LiDAR DEM (30 m) (a) and the Copernicus DEM (30 m) (b). Overall, the highest absolute error values are found in the southeastern and central Netherlands, particularly in areas with topographic features like hills. In contrast, the lowest absolute error values are observed in the delta regions of the Rhine, Meuse, and Scheldt rivers (see Fig. 16, and Fig. 17). Therefore, it can be concluded that the increase in terrain complexity leads to higher absolute error in the ASM derived from the Copernicus DEM. Additionally, the absolute error values for improved resolution (6 m) are lower than the absolute errors calculated for the original Copernicus-derived ASM. To further prove this claim, Fig. A.4 displays maps of absolute errors for the ASM derived from the Copernicus DEM (30 m) (a) as well as 6 m enhanced resolution by EDSR (b) respective to the ASM of LiDAR in the Limburg province located in the southern part of the Netherlands which includes relatively intense topographic changes in urban and non-urban areas. Also, Fig. A.3 displays the map of the 30 m LiDAR (a) and the Copernicus DEMs (b) of the mentioned area. By inspecting the error maps, it can be inferred that the EDSR super-resolution model has enhanced the resolution of ASM derived from the Copernicus DEM.

## 6 Further experiments and discussion

### 6.1 Comparative study

In this section, the results obtained from this research are compared with the findings of previous studies. Table 7 compares the results of the implemented super-resolution models in the literature for spatial resolution enhancement of solar energy maps. In this table, the performance of the Enhanced Super-Resolution GAN (ESRGAN), the Physics-Informed Resolution-Enhancing GAN (PHIREGAN), and the Super-Resolution Convolutional Neural Network (SRCNN) models are compared with the results of this study (EDSR model) using the evaluation metrics including; rMSE, PSNR, and SSIM.

**Table 7:** Comparing the results of the super-resolution models implemented in previous studies with the results of super-resolution by EDSR implemented in this study.

Study	Super-Resolution Model	Spatial Resolution of Solar Energy Map	Scale of Super Resolution	rMSE	PSNR (dB)	SSIM
This Study	EDSR	30 m	5	0.02	51.45	0.95
Karen Stengel et al. (2020)	PhIREGAN	100 km	25	0.08	-	-
Kurinchi-Vendhan (2021)	ESRGAN	20 km	5	-	31.82	0.56
Kurinchi-Vendhan et al. (2021)	SRCNN	20 km	5	-	29.05	0.52

The results demonstrate that the EDSR model, as implemented in this research, achieves the lowest rMSE, indicating higher accuracy than the SRCNN and GAN-based super-resolution models. Additionally, the PSNR value for the EDSR model is the highest, meaning it produces high-resolution patches with lower noise levels compared to SRCNN and GAN-based models. Furthermore, based on the SSIM metric, it can be concluded that the EDSR model results in patches with higher structural similarity to the reference images than those produced by the SRCNN and GAN-based models.

It is also worth mentioning that in this study, the super-resolution of solar energy potential maps was performed at a higher spatial resolution, whereas the studies mentioned in Table 7 focused on lower resolutions (100 km and 20 km). Since higher-resolution super-resolution requires more detailed reconstruction, the results of this research demonstrate the EDSR model's superior capability for super-resolving solar energy maps compared to SRCNN and GANs.

### 6.2 Placement of solar panels in urban areas

One of the most important applications of solar energy maps is the placement of solar panels in specific locations, such as building rooftops. For this purpose, high resolution maps are needed. In this study, by improving the resolution of the solar energy potential map derived from the Copernicus DEM from 30 m to 6 m using the EDSR model, the accuracy of this map has consequently increased. However, it is necessary to examine the usability of the produced 6 m map specifically for locating solar panels on rooftops in urban areas. In order to assess the feasibility and suitability of building rooftop for solar panel installation using the improved resolution map, a specific area in the center of Amsterdam, has been considered, and the building footprints have been obtained from the Open Street Map (OSM) platform.

First, the ASM, which is generated in $\frac{Wh}{m^{2}}$, is converted to $\frac{kWh}{m^{2}}$. Buildings with a roof area less than 30 square meters or an annual average solar energy received by the cells covering their roofs less than 800 $\frac{kWh}{m^{2}}$ are not economical for solar panel installation and therefore are excluded.

The Total Potential of Solar Radiation (TPSR) received by the roof of each building in 2021 is calculated in megawatt-hours per square meter ($\frac{MWh}{m^{2}}$) using the following eq.:

(14) $$TPSR(\frac{MWh}{m^{2}})=AAPSR(\frac{kWh}{m^{2}})\times Area(m^{2})\times 10^{-3},$$

where, Area denotes the roof area of each building in square meters, and AAPSR represents the Annual Average Potential of Solar Radiation received by the cells covering the roof of that building in $\frac{kWh}{m^{2}}$.

According to the standards set by the United States Environmental Protection Agency (EPA), solar panels convert 16% of incoming solar energy into electricity and are capable of retaining 86% of that electricity during installation [22]. Therefore, to calculate the Electric Power Production Potential (EPPP) by the panels installed on building rooftops in 2021 (in megawatt-hours per year), the following equation is used:

(15) EPPP=TPSR×0.86×0.16

**Figure 18:**
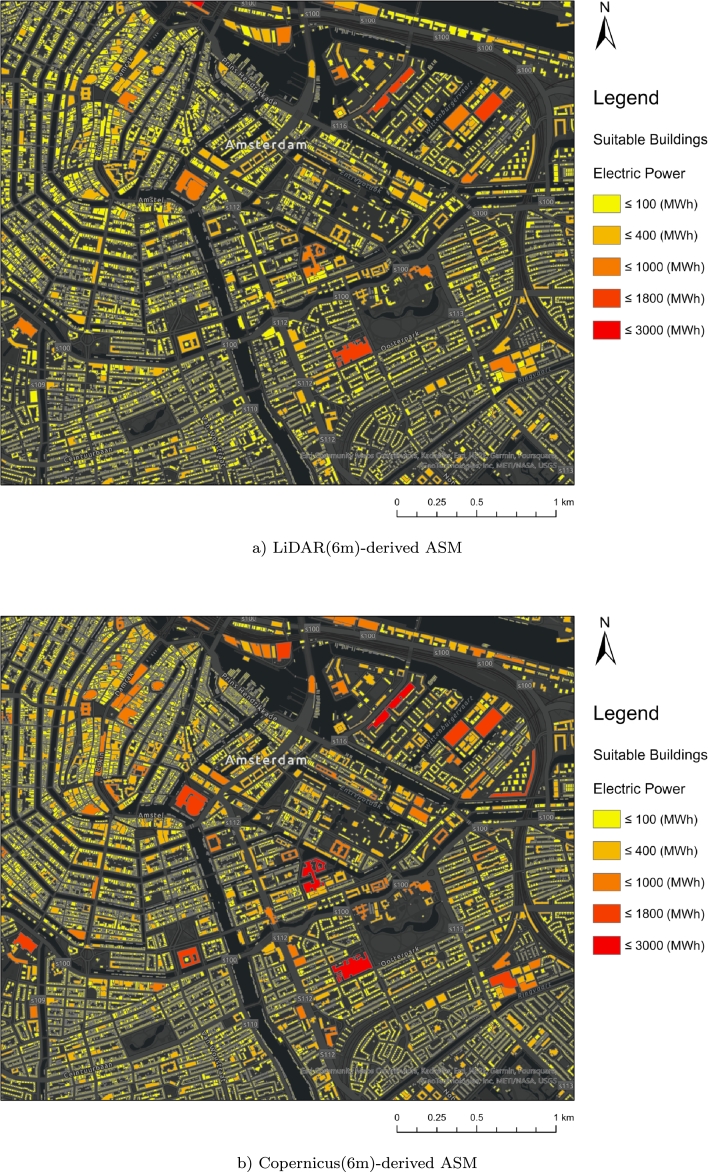
Maps of building suitability for installing solar panels based on the EPPP obtained from LiDAR 6 m DEM (a), and enhanced resolution of Copernicus ASM by EDSR (b).

**Figure 19:**
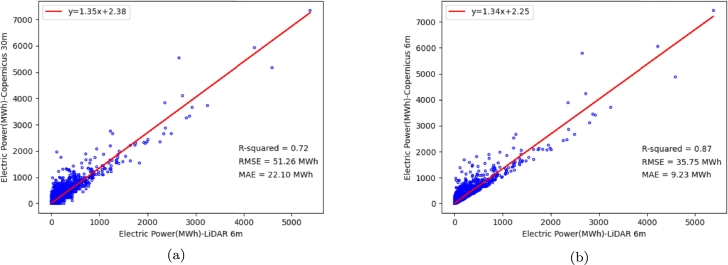
a) Scatter plot of the EPPP calculated from the Copernicus-based map of solar panel locating with a spatial resolution of 30 m versus the EPPP derived from the LiDAR map with a spatial resolution of 6 m, b) Scatter plot of the EPPP derived from the enhanced resolution (6 m) Copernicus against the EPPP generated from the LiDAR map (6 m).

In Fig. 18, the rooftop solar potential map of buildings for solar panel installation is displayed based on the EPPP. The map shows the EPPP values obtained from the LiDAR-derived ASM as well as the improved-resolution Copernicus ASM (6 m). The predicted annual EPPP values for suitable buildings in the Copernicus map range from 3 to 3890 $\frac{MWh}{year}$, while in the LiDAR map, the range is from 3 to 2805 $\frac{MWh}{year}$. In other words, the predicted annual EPPP for suitable buildings in the Copernicus map is higher by 1085 $\frac{MWh}{year}$ compared to the predicted EPPP in the LiDAR map. This indicates that the improved maps have overestimated solar energy potential for the buildings. However, as shown in Fig. 19, there is a good correlation between the LiDAR estimates and the improved map.

Fig. 19.a displays the scatter plot of the EPPP derived from the Copernicus-based map of building suitability for solar panel locating with a spatial resolution of 30 m compared to the EPPP achieved from the corresponding LiDAR map with a spatial resolution of 6 m. Similarly, Fig. 15.b depicts the scatter plot of EPPP achieved from the enhanced resolution Copernicus-based map of building suitability (6 m) compared to the EPPP calculated from the LiDAR map (6 m). The RMSE and MAE values for the high resolution Copernicus-derived EPPP, compared to the 6 m LiDAR high resolution, are 15.51 MWh and 1.66 MWh lower, respectively, than the RMSE and MAE values for the original Copernicus values (30 m) respective to the 6 m LiDAR-derived EPPP. Therefore, the improved-resolution Copernicus-derived EPPP exhibits higher accuracy compared to the 30 m resolution Copernicus EPPP. The R-squared value ($R^{2}$) for the high resolution Copernicus EPPP is 0.87, while for the original Copernicus EPPP is 0.72. This indicates that the high resolution Copernicus EPPP has a higher correlation with the 6 m LiDAR EPPP compared to the original Copernicus EPPP. Overall, it can be concluded that super-resolution, in addition to improving and enhancing the spatial resolution of the ASM derived from the Copernicus DEM, increases the accuracy and EPPP estimation.

Based on scatter plots, it can be concluded that the EPPP derived from both the high resolution and low resolution Copernicus ASM is somewhat prone to overestimation compared to the EPPP calculated from the LiDAR data. It is consistent with results achieved in previous studies that DEMs with low spatial resolution lead to overestimation in direct and general components of solar energy potential [2]. However, the magnitude of this overestimation has been reduced for high resolution Copernicus ASM compared to low resolution one. In other words, super-resolution, in addition to increasing and improving the spatial resolution of ASM derived from Copernicus DEM, has led to a reduction in overestimation in the EPPP estimation.

### 6.3 Performance assessment of super-resolution models for solar energy potential maps in areas with different land uses

The super-resolution of global DEMs from a spatial resolution of 30 m to a resolution of 6 m was performed in various land types in this study.

In non-urban areas, where the terrain is flat and there are no specific interfering factors, there is not a significant difference between ASMs with a spatial resolution of 30 m and 6 m. However, in non-urban areas with complex topography, such as the central and southeast parts of the Netherlands, which have hilly terrain, the ASM derived from Copernicus DEM with a spatial resolution of 6 m exhibits lower absolute errors compared to the ASM with a spatial resolution of 30 m (absolute error maps shown in Figs. 16.a and 16.b). In non-urban areas, the difference in RMSE between the ASMs derived from Copernicus DEMs with a spatial resolution of 6 m and 30 m is 2.478 $\frac{kWh}{m^{2}}$. This difference is mainly influenced by the complex topography of these areas.

In urban areas, the objective is to locate solar panel installations on building rooftops. These areas also exhibit significant variations in topography due to the presence of buildings with different heights, densely located in close proximity to each other. Therefore, the process of locating suitable positions for solar panel installations in urban areas requires higher precision and more detailed considerations. In urban areas, the difference in RMSE between the ASMs derived from Copernicus DEMs with a spatial resolution of 6 m and 30 m is 2.088 $\frac{kWh}{m^{2}}$. In other words, improving the spatial resolution of the Copernicus-derived ASM leads to improved accuracy. Additionally, as shown in scatter plots (Figs. 19.a and 19.b), the improvement in spatial resolution enhances the correlation between the EPPP derived from the Copernicus-based ASM and the EPPP derived from the LiDAR-based ASM.

The highway-adjacent areas are typically underutilized public lands, with convenient access to power transmission lines and fewer objects that could result in reduced shading and increased solar energy potential for the panels. Consequently, the costs associated with land acquisition for solar panel installation and energy transmission can be eliminated or reduced. Thus, these areas are suitable for solar panel installations. However, highways have identified margins or setbacks. Therefore, improving the spatial resolution of ASMs along highway margins greatly assists in the precise placement of solar panels. In the highway margin areas, the difference in RMSE between the ASMs derived from Copernicus DEMs with a spatial resolution of 6 m and 30 m is 1.652 $\frac{kWh}{m^{2}}$.

### 6.4 Generalization and transfer learning

In this research, five times of super-resolution improvement have been applied to the ASMs, transitioning from a 30 m to a 6 m spatial resolution. Consequently, the output ASMs possess a spatial resolution of 6 m, which corresponds to a lower level of detail in building structures (Level of Detail 1, LOD1). Therefore, it appears that the model possesses generalizability for areas with different urban morphological structures. However, in order to enhance the model's performance in new study areas, it is recommended to fine-tune the model using transfer learning techniques with a limited amount of training data. Although, in this study, the model was initially trained on high resolution LiDAR-based ASMs with 6 m and 30 m spatial resolutions, and then the trained model by LiDAR data was transferred to improve the spatial resolution of Copernicus-derived ASM with a 30 m resolution in various urban areas, non-urban areas, and highway margins. Considering the favorable results and improvements achieved by enhancing the spatial resolution of Copernicus ASM, it is plausible to infer the model's generalizability. Consequently, this developed model can be utilized to improve the spatial resolution of ASMs in other target areas where LiDAR data is unavailable.

### 6.5 Limitations and suggestions

Various approaches can be proposed to improve spatial resolution. One key aspect of this research is enhancing the spatial resolution of ASMs derived from the Copernicus DEM rather than improving the spatial resolution of the DEM itself and subsequently generating the improved ASMs from the enhanced DEM. Improving the resolution of the DEM is more challenging and complex than improving the resolution of solar energy maps [19]. In improving the resolution of the DEM, it is essential to enhance the resolution and improve the DEM quality in areas with significant topographic variations, such as ridges and valleys, which may not be of great importance in estimating solar radiation and generating solar energy potential maps. Additionally, using high-resolution DEMs to generate solar energy potential maps with physical models like area solar radiation incurs significant computational and time costs. Therefore, utilizing medium-resolution DEMs and then applying super-resolution techniques, such as those used in this study, can help reduce both computational and time expenses while still providing improved spatial resolution for the generated maps. Moreover, while numerous studies on DEM super-resolution have predominantly concentrated on natural landscapes such as ridges and valleys, urban environments—critical for solar panel installations—are often neglected [33], [39]. Addressing this gap presents a promising direction for future research. Applying super-resolution techniques to DEMs in urban areas is essential for generating high-resolution maps of solar energy potential, which are instrumental in optimizing rooftop solar panel placement. This research focus could significantly advance efforts to enhance the efficiency of solar energy systems in urban settings.

In this study, single-image super-resolution (SISR) methods were utilized, where the employed super-resolution models received a low resolution (30 m) image patch of a LiDAR-derived ASM as input and a corresponding high resolution generated (6 m) image patch as output. The single-image super-resolution method employed a supervised learning method, thereby requiring pairs of image patches of ASMs with low and corresponding high spatial resolution during the learning process. However, it is possible to employ multi-image super-resolution (MISR) methods based on unsupervised learning method. These methods utilize multiple low resolution image patches of ASMs to generate corresponding high resolution image patches, eliminating the need for high resolution image patches of the ASM during the learning process [68].

Moreover, the proposed model for enhancing spatial resolution is initially trained based on paired image patches of the low resolution (30 m) and high resolution (6 m) LiDAR-derived ASM. Then, using transfer learning, the model is utilized to improve the spatial resolution of image patches from the Copernicus-derived ASM with a resolution of 30 m to generate an ASM with a spatial resolution of 6 m. While, it is possible to generate image patches of the Copernicus-derived ASM with spatial resolution of 150 m from the corresponding image patches with spatial resolution of 30 m using resampling methods. Then, the super-resolution model is trained on this data. Then, the trained model is employed to enhance the spatial resolution of image patches from the Copernicus-derived ASM with a spatial resolution of 30 m for five times, resulting in the generation of corresponding image patches with a spatial resolution of 6 m. Consequently, the super-resolution model eliminates the need for high resolution ASMs and mitigates financial, computational, and accessibility challenges associated with high resolution LiDAR DEM. However, in this way, the ASM overestimation may not be improved and will also remain at the enhanced-resolution ASM.

In this study, the OSM voluntary geographic information was used to obtain building footprints. However, the OSM data may not be synchronized with the year of producing the ASM and the year of generating the LiDAR and Copernicus DEMs. Therefore, it might be better to consider using alternative mechanisms to automatically obtain building footprints.

One of the reasons for the over estimation of EPPP in the estimation of the high resolution Copernicus data is the training of the super-resolution model based on the LR and HR ASM patches obtained from the LiDAR DEM. The lack of super-resolution model training based on the LR data of the Copernicus-derived ASM (30 m) and the corresponding HR data of LiDAR-derived ASM (6 m), can be considered as a limitation of this research. However, the super-resolution procedure becomes dependent to the high resolution samples which may decrease the generalization of the model.

Super-resolution of medium resolution DEMs is particularly crucial in non-urban areas, especially when analyzing time series data (monthly, daily, etc.), as they allow for more accurate and faster estimations of solar energy potential. They offer a cost-effective alternative to physically-based energy estimation models, which have higher computational burdens. By considering meteorological parameters such as cloud cover and sunshine hours, more precise calculations of the electricity generated from the received solar energy by the panels can be realized. This leads to more accurate planning of the energy output from solar panels and holds implications for future energy management strategies.

## 7 Conclusion

As the global population continues to grow, the energy demand is increasing. Due to the finite nature of non-renewable energy resources like fossil fuels, oil, and gas, along with the environmental challenges posed by their use, renewable energy sources, particularly solar energy, are gaining significant attention. To effectively harness solar energy, precise site selection for solar panel installations in diverse areas (urban, non-urban, and highway peripheries) is essential. This requires accurate solar energy potential mapping, which can be achieved through solar radiation estimation models using DEMs. The accuracy and resolution of these DEMs are critical in determining the precision of solar energy potential maps. However, obtaining high resolution DEMs, such as those generated by LiDAR, on a global scale is not feasible due to the high costs and significant storage requirements associated with LiDAR data. In response, global DEMs with open access offer a practical alternative for generating solar energy potential maps that are accessible to the public. However, these maps typically have a resolution limit of 30 m, which is insufficient for optimizing solar panel placement, especially on building rooftops in urban areas. Deep learning-based super-resolution techniques present a promising solution to this limitation. This research proposes a framework that leverages these techniques—specifically, the EDSR model and a U-Net-based super-resolution model with various backbones such as ResNet 18, 34, 50, and 101—to achieve a five-fold improvement in the spatial resolution of solar energy potential maps derived from global DEMs. These models enhance the spatial resolution, facilitating more accurate solar panel placement, particularly in urban settings. The study begins by training and testing these models on high resolution (6 m) and low resolution (30 m) solar energy potential maps derived from LiDAR DEMs (from the year 2021). After assessing the accuracy of these super-resolution models, the EDSR model emerged as the most accurate and stable, playing a key role in the research. Subsequently, the accuracy of solar energy potential maps derived from various global DEMs was evaluated. The Copernicus DEM was selected due to its superior performance in urban areas. The EDSR super-resolution model was then applied to enhance the resolution of the Copernicus DEM-derived solar energy potential map from 30 m to 6 m. A comparative analysis between the Copernicus DEM-based map and the EDSR-enhanced map, using a LiDAR-based reference, demonstrated that the EDSR model not only improved the map's resolution but also enhanced the accuracy of solar energy estimation, particularly in urban areas and along major highways. Finally, the improved 6-meter resolution map was assessed for its effectiveness in identifying suitable buildings for solar panel installation. The results showed that the enhanced map had a high potential for accurately determining optimal locations for rooftop solar panels, thus contributing to more efficient solar energy utilization.

## Funding statement

No funding is available for this work.

## CRediT authorship contribution statement

**Maryam Hosseini:** Writing – original draft, Visualization, Validation, Software, Investigation, Data curation. **Hossein Bagheri:** Writing – review & editing, Supervision, Software, Methodology, Investigation, Conceptualization.

## Declaration of Competing Interest

The authors declare that they have no known competing financial interests or personal relationships that could have appeared to influence the work reported in this paper.

## Data Availability

The data that support the findings of this study are available from the corresponding author, [H.B.], upon reasonable request.
